# Nonparametric Neighborhood Selection in Graphical Models

**Published:** 2022

**Authors:** Hao Dong, Yuedong Wang

**Affiliations:** Department of Statistics and Applied Probability, University of California, Santa Barbara, Santa Barbara, CA, USA; Department of Statistics and Applied Probability, University of California, Santa Barbara, Santa Barbara, CA, USA

**Keywords:** conditional density estimation, mixed data, regularization, reproducing kernel Hilbert space, smoothing spline ANOVA

## Abstract

The neighborhood selection method directly explores the conditional dependence structure and has been widely used to construct undirected graphical models. However, except for some special cases with discrete data, there is little research on nonparametric methods for neighborhood selection with mixed data. This paper develops a fully nonparametric neighborhood selection method under a consolidated smoothing spline ANOVA (SS ANOVA) decomposition framework. The proposed model is flexible and contains many existing models as special cases. The proposed method provides a unified framework for mixed data without any restrictions on the type of each random variable. We detect edges by applying an $L_{1}$ regularization to interactions in the SS ANOVA decomposition. We propose an iterative procedure to compute the estimates and establish the convergence rates for conditional density and interactions. Simulations indicate that the proposed methods perform well under Gaussian and non-Gaussian settings. We illustrate the proposed methods using two real data examples.

## 1. Introduction

Discovering conditional independence among random variables is an essential task in statistics. Undirected probabilistic graphical models play a pivotal role in characterizing conditional independence. They have been utilized in a wide range of scientific and engineering domains, including statistical physics, computer vision, machine learning, and computational biology (Koller and Friedman, 2009). A graphical model is constructed based on an undirected graph G=(V,E) with node set V={1,⋯,p} representing p random variables $X_{1},\cdots ,X_{p}$ and edge set E⊆V×V describing the conditional dependence among $X_{1},\cdots ,X_{p}$. Let $X=(X_{1},\cdots ,X_{p})$ and $X_{\setminus \{i_{1},\cdots ,i_{k}\}}$ be the sub-vector of X without elements in $\left\{i_{1},\cdots ,i_{k}\right\}$. Then, {i,j}∉E corresponds to the conditional independence between $X_{i}$ and $X_{j}$ given other variables in X, denoted as $X_{i}⊥X_{j}|X_{\setminus \{i,j\}}$.

As joint density ultimately determines the conditional relationship, methods for edge detection based on estimating joint density have been proposed (Yuan and Lin, 2007; Banerjee et al., 2008; Friedman et al., 2008; Hsieh et al., 2014; Liu et al., 2009). Under the Gaussian assumption of X∼𝒩(0,Σ), the task of edge detection reduces to the estimation of the precision matrix $\Sigma^{-1}$. Yuan and Lin (2007), Banerjee et al. (2008), and Friedman et al. (2008) proposed regularization methods that minimize the log-likelihood with an $L_{1}$ penalty on the entries of $\Sigma^{-1}$. Hsieh et al. (2014) proposed a fast second-order algorithm for solving the $L_{1}$-regularized Gaussian MLE. Liu et al. (2009) extended the $L_{1}$-regularized Gaussian MLE approach to the setting where there exist monotone transformations $f_{1},\cdots ,f_{p}$ such that $\left(f_{1}(X_{1}),\cdots ,f_{p}(X_{p}))∼𝒩(0,\Sigma \right)$. These parametric and semi-parametric methods may be too restrictive for some applications and cannot handle mixed data since they rely on the Gaussian assumption.

Let f(x) be the joint density function of X, and consider the transformation $f(x)=e^{\eta (x)}∕\int e^{\eta (x)}dx$, where η(x) is the logistic transformation of f. The SS ANOVA decomposition represents η(x) as a summation of a constant, main effects, and interactions:

(1) $$\eta (x_{1},\cdots ,x_{p})=c+\sum_{j=1}^{p}\eta_{j}(x_{j})+\sum_{1\leq j<k\leq p}\eta_{jk}(x_{j},x_{k})+\cdots +\eta_{1\cdots p}(x_{1},\cdots ,x_{p}).$$

The conditional independence $X_{j}⊥X_{k}|X_{\setminus \{j,k\}}$ is equivalent to the summation of all interactions involving $x_{j}$ and $x_{k}$ equal to zero (Gu, 2013). Consequently, identifying edges is equivalent to identifying nonzero interactions. Jeon and Lin (2006) developed a penalized M-estimation method for edge detection based on the SS ANOVA decomposition (1). Our experience indicates that this joint density estimation approach is only computationally feasible with a small p due to large memory requirements.

The neighborhood selection approach explores structures in conditional densities and is usually more computationally efficient. By the conditional independence properties of undirected graphical models, for any node α∈V, $X_{\alpha }$ only depends on other variables in its neighborhood set $nb_{G}(\alpha )$, where $nb_{G}(\alpha )=\{k\in V|\{\alpha ,k\}\in E\}$. Consequently, the conditional independence structure of graph G can be constructed by estimating all of its neighborhoods $nb_{G}(\alpha )$ for α=1,⋯,p. Neighborhood selection aims to identify a minimal set of variables $nb_{G}(\alpha )$ that $X_{\alpha }$ depends on for each node α∈V.

Many neighborhood selection methods have been developed for learning sparse graphical models (Hastie et al., 2015; Drton and Maathuis, 2017). Flexible models were proposed for discrete data (Höfling and Tibshirani, 2009; Ravikumar et al., 2010). Methods for continuous data usually model the conditional mean (Meinshausen and Bühlmann, 2006; Voorman et al., 2014) or conditional quantiles (Ali et al., 2016). For example, Meinshausen and Bühlmann (2006) and Peng et al. (2009) considered a linear model for the conditional mean with $L_{1}$ penalties on coefficients and partial correlations, respectively. Voorman et al. (2014) considered an additive model for the conditional mean. The conditional mean approach does not assume a specific distribution for the regression error and therefore appears to be distribution-free. However, if the conditional relationships are linear, the joint distribution must be multivariate Gaussian under mild assumptions (Voorman et al., 2014). In other words, the restriction of Gaussianity has not been removed as it appears. For mixed continuous and discrete variables, Lee and Hastie (2015) considered a pairwise model that generalizes Gaussian graphical and discrete models. Chen et al. (2015) proposed a flexible pairwise graphical model where each node’s conditional distribution is in the exponential family. Gu and Ma (2011) developed a functional ANOVA method for estimating the conditional density of cross-classified responses and identifying conditional independence structure through Kullback-Leibler projection.

Modeling mixed data is challenging since it is difficult to specify a joint density. Existing neighborhood selection methods are restrictive since they model specific mixed data or assume specific conditional distributions. In this paper, we propose a new fully non-parametric neighborhood selection method. We construct an SS ANOVA model for each conditional density and select neighborhood via $L_{1}$ regularization. The new contributions of our neighborhood selection method consist of four parts. First, we directly target the neighborhood definition based on conditional density without assuming any specific family of distributions. The whole conditional density provides the most comprehensive summary of the relationship which might be missed by specific characteristics such as conditional mean and quantiles. Second, we allow the range of each random variable to be an arbitrary set and use the tensor product of reproducing kernel Hilbert spaces (RKHS) to construct a model space for each conditional density. Therefore, the proposed method provides a unified framework for mixed data types without any restrictions on the type of each random variable. The proposed model is more general and flexible than existing models. Third, we use the SS ANOVA structure to facilitate the selection of neighborhoods. Specifically, we estimate the conditional density for each node based on SS ANOVA decomposition with an $L_{1}$ penalty involving interaction components. This approach for neighborhood selection has not been studied before. Last but not least, the new neighborhood selection method based on conditional density is more computationally efficient than those based on joint density and is parallelizable.

The rest of the paper is organized as follows. Section 2 introduces the new neighborhood selection method. Section 3 presents the computational method and the implementation of the proposed algorithm. Section 4 derives the convergence rate of the conditional density estimate and its components in the SS ANOVA decomposition. Section 5 conducts simulations to compare edge detection performance with existing methods under both Gaussian and non-Gaussian settings. Section 6 illustrates the proposed methods using two real data sets. Section 7 provides some discussion. The Appendix contains proofs and auxiliary material.

## 2. Neighborhood Selection Through Conditional Density Estimation with $L_{1}$ Penalty

In this section, we first introduce some notation and the SS ANOVA decomposition. Then, we present our nonparametric method for edge detection.

### 2.1 Notation and SS ANOVA Decomposition

Consider p random variables $X_{1},\cdots ,X_{p}$ with ranges denoted as ${𝒳}_{1},\cdots ,{𝒳}_{p}$. Each range ${𝒳}_{\alpha }$ is an arbitrary set for generality. It may be a continuous interval, a discrete set, or a circle. It could even be a subset in Euclidean space or a sphere. That is, each ${𝒳}_{\alpha }$ could be a multivariate random variable. Denote $X=(X_{1},\cdots ,X_{p})$ as the p-dimensional random vector with range $𝒳={𝒳}_{1}\times \cdots \times {𝒳}_{p}$ and $x=(x_{1},\cdots ,x_{p})$ as a realization of the random vector. For a fixed α∈V={1,⋯,p}, denote $X_{\setminus \{\alpha \}}=(X_{1},\cdots ,X_{\alpha -1},X_{\alpha +1},\cdots ,X_{p})$ and $x_{\setminus \{\alpha \}}=(x_{1},\cdots ,x_{\alpha -1},x_{\alpha +1},\cdots ,x_{p})$ as the vectors of X and x with the αth element being removed. Our goal is to select the neighborhood $nb_{G}(\alpha )$ through the estimation of the conditional density $f(x_{\alpha }|x_{\setminus \{\alpha \}})$.

Denote $X_{i}=(X_{i,1},\cdots ,X_{i,p})$ and $x_{i}=(x_{i,1},\cdots ,x_{i,p})$ for i=1,⋯,n as n i.i.d. random vectors and their realizations. Let $x_{i,\setminus \{\alpha \}}=(x_{i,1},\cdots ,x_{i,\alpha -1},x_{i,\alpha +1},\cdots ,x_{i,p})$. Denote $x_{i}^{\alpha }=(x_{i,1},\cdots ,x_{i,\alpha -1},x_{\alpha },x_{i,\alpha +1},\cdots ,x_{i,p})$ as the p-dimensional vector with $x_{\alpha }$ varies in ${𝒳}_{\alpha }$ and all other variables fixed at their ith realizations. For simplicity, the dependence of $x_{i}^{\alpha }$ on $x_{\alpha }$ is not expressed explicitly.

Let ${ℋ}^{\left(j\right)}$ be an RKHS on ${𝒳}_{j}$ and ${ℋ}^{\left(j\right)}=\{1_{\left(j\right)}\}⊕{ℋ}_{\left(j\right)}$, where $\left\{1_{\left(j\right)}\right\}$ is the space of the constant functions on ${𝒳}_{j}$ and ${ℋ}_{\left(j\right)}$ is the orthogonal complement of $\left\{1_{\left(j\right)}\right\}$. One may construct a flexible and interpretable model for a p-dimensional function through the following SS ANOVA decomposition of the tensor product space $\overset{p}{\underset{j=1}{⊗}}{ℋ}^{\left(j\right)}$ on 𝒳 (Wang, 2011; Gu, 2013):

(2) $$\overset{p}{\underset{j=1}{⊗}}{ℋ}^{\left(j\right)}=\overset{p}{\underset{j=1}{⊗}}\{\{1_{\left(j\right)}\}⊕{ℋ}_{\left(j\right)}\}\;\;=\{1\}⊕\left\{\overset{p}{\underset{j=1}{⊕}}{ℋ}_{\left(j\right)}\right\}⊕\left\{\underset{1\leq j<k\leq p}{⊕}[{ℋ}_{\left(j\right)}⊗{ℋ}_{\left(k\right)}]\right\}⊕\cdots ⊕\{{ℋ}_{\left(1\right)}⊗\cdots ⊗{ℋ}_{\left(p\right)}\}.$$

The decomposition in equation (1) corresponds to the SS ANOVA decomposition to the logistic transformation of the joint density function.

### 2.2 SS ANOVA Model for Conditional Density

For the conditional density of $X_{\alpha }$, we consider the logistic density transformation

(3) $$f(x_{\alpha }|x_{\setminus \{\alpha \}})=\frac{e^{\eta (x)}}{\int_{{𝒳}_{\alpha }}e^{\eta (x)}dx_{\alpha }}$$

to enforce the conditions of f>0 and ∫f=1. The function η is the logistic transformation of f. An SS ANOVA model for η in (3) may contain any subset of components in the SS ANOVA decomposition (2). For simplicity, we assume that $\eta \in {ℳ}_{\alpha }$ where

(4) $${ℳ}_{\alpha }=\{1\}⊕\left\{\overset{p}{\underset{j=1}{⊕}}{ℋ}_{\left(j\right)}\right\}⊕\left\{\underset{k\neq \alpha }{⊕}[{ℋ}_{\left(\alpha \right)}⊗{ℋ}_{\left(k\right)}]\right\}$$

is a subspace with main effects and two-way interactions only. A function $\eta \in {ℳ}_{\alpha }$ can be decomposed as follows:

(5) $$\eta (x)=\varsigma +\sum_{j=1}^{p}\eta_{j}(x_{j})+\sum_{k\neq \alpha }\eta_{\alpha k}(x_{\alpha },x_{k}),$$

where each functional component in (5) belongs to the corresponding subspace in (4). We note that the proposed method can be easily extended to include higher-order interactions.

**Remark 1** The SS ANOVA model (1) for the joint density with main effects and two-way interaction only is a pairwise graphical model, which is commonly assumed in the existing literature.

**Remark 2** We consider both the log-likelihood and pseudo log-likelihood approaches for estimating the conditional density (Gu, 2013). We present the pseudo likelihood approach in the main text since it is computationally more efficient. The log-likelihood approach is presented in Appendix A. For the pseudo log-likelihood approach, model space ${ℳ}_{\alpha }$ includes constant functions. The model space for the log-likelihood approach eliminates the constant functions for identifiability.

**Remark 3** To compare the estimation between the joint and neighborhood approaches under pairwise graphical models, we consider the SS ANOVA decomposition (1) with main effects and two-way interaction only for the joint density and the SS ANOVA decomposition (5) for the conditional density. The joint density approach needs to estimate all main effects and two-way interactions simultaneously with a total number of components proportional to $p^{2}$. Our experience indicates that this joint approach is computationally infeasible even with moderately large p due to memory constraints. On the other hand, the neighborhood approach needs to estimate p main effects and p−1 two-way interactions for each node, which significantly reduces the computational cost and memory requirement and is parallelizable.

**Remark 4** Model (5) contains many parametric models as special cases. Specifically, the Gaussian graphical model is a special case with ${𝒳}_{j}=R$, $\eta_{j}(x_{j})=\beta_{j}x_{j}-x_{j}^{2}∕2$ for j=α and 0 otherwise, and $\eta_{\alpha k}(x_{\alpha },x_{k})=\beta_{\alpha k}x_{\alpha }x_{k}$ for some constants $\beta_{j}$ and $\beta_{\alpha k}$. The Ising model for binary data is a special case with ${𝒳}_{j}=\{0,1\}$, $\eta_{j}(x_{j})=x_{j}$ for j=α and 0 otherwise, and $\eta_{\alpha k}(x_{\alpha },x_{k})=\beta_{\alpha k}x_{\alpha }x_{k}$. The Poisson graphical model for discrete data is a special case with ${𝒳}_{j}=\{0,1,2,\cdots \}$, $\eta_{j}(x_{j})=x_{j}-\log(x_{j}!)$ for j=α and 0 otherwise, and $\eta_{\alpha k}(x_{\alpha },x_{k})=\beta_{\alpha k}x_{\alpha }x_{k}$. The exponential family model proposed by Suggala et al. (2017),

(6) $$\log f(x_{\alpha }|x_{\setminus \{\alpha \}})\propto \left\{\beta_{\alpha }B_{\alpha }(x_{\alpha })+\sum_{\{\alpha ,k\}\in E}\beta_{\alpha k}B_{\alpha }(x_{\alpha })B_{k}(x_{k})+C_{\alpha }(x_{\alpha })\right\},$$

is also a special case with $\eta_{j}(x_{j})=\beta_{j}B_{j}(x_{j})+C_{j}(x_{j})$ for j=α and 0 otherwise, and $\eta_{\alpha k}(x_{\alpha },x_{k})=\beta_{\alpha k}B_{\alpha }(x_{\alpha })B_{k}(x_{k})$. Note that many existing exponential family models including (6) assume a multiplicative interaction while model (5) does not assume any specific interaction. Therefore, the proposed model is more general.

### 2.3 Penalized Pseudo Log-likelihood Estimation

For each node α∈V, we assume that $\eta (x)\;\in \;{ℳ}_{\alpha }$ where ${ℳ}_{\alpha }$ is given in (4) and η is decomposed as in (5). We further decompose ${ℋ}_{\left(j\right)}$ as ${ℋ}_{\left(j\right)}={ℋ}_{\left(j\right)}^{0}⊕{ℋ}_{\left(j\right)}^{1}$ where ${ℋ}_{\left(j\right)}^{0}$ is a finite dimensional space containing functions that are not subject to penalty. We estimate η in (5) by minimizing the following penalized pseudo log-likelihood in ${ℳ}_{\alpha }$:

(7) $$l_{\alpha }+\frac{\lambda_{1}}{2}\sum_{j=1}^{p}\theta_{j}^{-1}{\left\|P_{j}\eta_{j}\right\|}^{2}+\tau_{1}\sum_{k\neq \alpha }w_{\alpha k}\|\eta_{\alpha k}\|,$$

where $l_{\alpha }=n^{-1}\sum_{i=1}^{n}\left\{e^{-\eta (x_{i})}+\int_{{𝒳}_{\alpha }}\eta (x_{i}^{\alpha })\rho (x_{i}^{\alpha })dx_{\alpha }\right\}$ is the pseudo log-likelihood, ρ(⋅) is a known density of $X_{\alpha }$ conditional on $X_{\setminus \{\alpha \}}=x_{i,\setminus \{\alpha \}}$, $P_{j}$ is the projection operator onto ${ℋ}_{\left(j\right)}^{1}$, $\lambda_{1}$, $\tau_{1}$, and $\theta_{j}’s$ are tuning parameters, $0\leq w_{\alpha k}<\infty$ are pre-specified weights, and ‖⋅‖ is an induced norm in ${ℳ}_{\alpha }$. The pseudo log-likelihood $l_{\alpha }$ measures the goodness-of-fit. The second element in (7) is the roughness $L_{2}$ penalty on main effects. The third element in (7) is the $L_{1}$ penalty for selecting the neighborhood $nb_{G}(\alpha )$. We allow different weights in the $L_{1}$ penalty for flexibility.

**Remark 5** The idea of pseudo log-likelihood was first developed in Jeon and Lin (2006) for joint density estimation. Gu (2013) extended this approach to conditional density estimation. We present the pseudo log-likelihood estimation in the main text since this approach is computationally more efficient. The log-likelihood approach to conditional density estimation needs to calculate the integral $\int_{{𝒳}_{\alpha }}e^{\eta (x_{i}^{\alpha })}dx_{\alpha }$ repeatedly, which can be computationally intensive. With a proper choice of ρ, the pseudo log-likelihood approach needs to calculate an integral only once.

**Remark 6** The proposed method replaces the $L_{2}$ penalty on interactions in Gu (2013) with the $L_{1}$ penalty for neighborhood selection and differs from that in Jeon and Lin (2006) in two aspects. First, Jeon and Lin (2006)’s approach is a global method that estimates the joint density; thus is computationally intensive and can only handle small dimensions p. Second, Jeon and Lin (2006) posed the $L_{1}$ penalty to both main effects and interactions. Consequently, their method selects both nodes and edges. In practice, the nodes are usually given, and the goal is to detect edges. Therefore, we consider the smoothness promoting $L_{2}$ penalty to main effects and the sparsity promoting $L_{1}$ penalty to interactions.

Let

(8) $$𝒢=\left\{\overset{p}{\underset{j=1}{⊕}}{ℋ}_{\left(j\right)}\right\}⊕\left\{\underset{k\neq \alpha }{⊕}[{ℋ}_{\left(\alpha \right)}⊗{ℋ}_{\left(k\right)}]\right\}.$$

We can rewrite η(x)=ς+g(x) where $g(x)=\sum_{j=1}^{p}\eta_{j}(x_{j})+\sum_{k\neq \alpha }\eta_{\alpha k}(x_{\alpha },x_{k})\in 𝒢$. We first estimate ς with fixed g and then estimate g using the profiled pseudo log-likelihood. The results are summarized in the following proposition.

**Proposition 1** *With fixed* g*, the minimizer of* ς *in* (7) *is* $\hat{\varsigma }=\log\{n^{-1}\sum_{i=1}^{n}e^{-g(x_{i})}\}$ *and the penalized pseudo log-likelihood* (7) *reduces to the following penalized profiled pseudo log-likelihood*

(9) $$l(\hat{\varsigma }(g),g)+\frac{\lambda_{1}}{2}\sum_{j=1}^{p}\theta_{j}^{-1}{\left\|P_{j}\eta_{j}\right\|}^{2}+\tau_{1}\sum_{k\neq \alpha }w_{\alpha k}\|\eta_{\alpha k}\|,$$

*where* $l(\hat{\varsigma }(g),g)=log\{n^{-1}\sum_{i=1}^{n}e^{-g(x_{i})}\}+n^{-1}\sum_{i=1}^{n}\int_{{𝒳}_{\alpha }}g(x_{i}^{\alpha })\rho (x_{i}^{\alpha })dx_{\alpha }$ *is the profiled pseudo log-likelihood*.

The proof can be found in Appendix C. Instead of minimizing (9) that involves $L_{1}$ penalties on functions, as in Lin and Zhang (2006), we will solve an equivalent but more convenient minimization problem that involves $L_{1}$ penalties on the smoothing parameters.

**Proposition 2** *Minimizing*

(10) $$l(\hat{\varsigma }(g),g)+\frac{\lambda_{1}}{2}\left(\sum_{j=1}^{p}\theta_{j}^{-1}{\left\|P_{j}\eta_{j}\right\|}^{2}+\sum_{k\neq \alpha }w_{\alpha k}\theta_{\alpha k}^{-1}{\left\|\eta_{\alpha k}\right\|}^{2}\right)+\lambda_{2}\sum_{k\neq \alpha }w_{\alpha k}\theta_{\alpha k},$$

*subject to* $\theta_{\alpha k}\geq 0$ *for* k=1,⋯,p *and* k≠α *is equivalent to minimizing* (9).

The proof of equivalence can be found in Appendix C. Proposition 2 transforms the selection of nonzero functions $\eta_{\alpha k}$ in (9) into a selection of nonzero parameters $\theta_{\alpha k}$. The minimization problem (10) consists of $L_{2}$ penalties on functions and $L_{1}$ penalties on parameters and existing methods can be modified to solve each part. Computational details for solving (10) are presented in Section 3.

Since the pseudo log-likelihood is used for estimation, we need to compute the conditional density estimate using the following proportion.

**Proposition 3** *The resulting estimate of the conditional density is* $\hat{f}(x_{\alpha }|x_{\setminus \{\alpha \}})\propto e^{\hat{g}(x)}\rho (x)$ *where* $\hat{g}$ *is the minimizer of* (10).

The proof of Proposition 3 is given in Appendix C. Notice that the minimization problem (10) involves p−1 two-way interaction terms. Solving (10) for all α=1,⋯,p leads to two estimates for each two-way interaction, denoted as ${\hat{\eta }}_{\alpha k}$ and ${\hat{\eta }}_{k\alpha }$ for α, k=1,⋯,p and α≠k. There are two commonly used rules to combine the results: AND-rule ({α,k}∈E iff ${\hat{\eta }}_{\alpha k}\neq 0$ and ${\hat{\eta }}_{k\alpha }\neq 0$) or OR-rule ({α,k}∈E iff ${\hat{\eta }}_{\alpha k}\neq 0$ or ${\hat{\eta }}_{k\alpha }\neq 0$) (Hastie et al., 2015). As discussed in Section 4.2 in Chen et al. (2015), when the αth and kth nodes are of the same type (same marginal distribution) or are both non-Gaussian, there is no clear reason to prefer one edge estimate over the other. We adopt the AND-rule in all simulations and real data examples.

## 3. Algorithm

In this section, we propose a computational algorithm that solves (10) iteratively. Denote $\theta_{1}={\left(\theta_{1},\cdots ,\theta_{p}\right)}^{T}$, $\theta_{2}={\left(\theta_{\alpha 1},\cdots ,\theta_{\alpha (\alpha -1)},\theta_{\alpha (\alpha +1)},\cdots ,\theta_{\alpha p}\right)}^{T}$, and $w={\left(w_{\alpha 1},\cdots ,w_{\alpha (\alpha -1)},w_{\alpha (\alpha +1)},\cdots ,w_{\alpha p}\right)}^{T}$. Let ${ℋ}_{\left(j\right)}={ℋ}_{\left(j\right)}^{0}⊕{ℋ}_{\left(j\right)}^{1}$ where ${ℋ}_{\left(j\right)}^{0}$ is a finite-dimensional space containing functions that are not subject to $L_{2}$ penalty. Denote $\phi_{j1},\cdots ,\phi_{jm_{j}}$ as basis functions of ${ℋ}_{\left(j\right)}^{0}$, and $R_{j}^{1}$, $R_{j}$, and $R_{\alpha k}$ as reproducing kernels of ${ℋ}_{\left(j\right)}^{1}$, ${ℋ}_{\left(j\right)}$, and ${ℋ}_{\left(\alpha k\right)}$, respectively. We collect all basis functions $\phi_{jk}$ for j=1,⋯,p and $k=1,\cdots ,m_{j}$ and denote them as $\phi ={\left(\phi_{1},\cdots ,\phi_{m}\right)}^{T}$, a vector of functions of x with dimension $m=\sum_{j=1}^{p}m_{j}$.

Since in general the minimization problem (10) does not have a solution in a finite-dimensional space, as in Gu (2013), we approximate the solution by a subset of representers. Specifically, let $\left\{{\tilde{x}}_{u}=({\tilde{x}}_{u,1},\cdots ,{\tilde{x}}_{u,p}),\;u=1,\cdots ,q\right\}$ be a subset of all observations $\left\{x_{i},\;i=1,\cdots ,n\right\}$. Let $\xi_{1ju}(x_{j})=R_{j}^{1}({\tilde{x}}_{u,j},x_{j})$ and $\xi_{\alpha ku}(x_{\alpha },x_{k})=R_{\alpha k}(({\tilde{x}}_{u,\alpha },{\tilde{x}}_{u,k}),(x_{\alpha },x_{k}))$ for u=1,⋯,q, k=1,⋯,p, and k≠α. Let $\xi_{\theta_{1},u}(x)=\sum_{j=1}^{p}\theta_{j}\xi_{1ju}(x_{j})$, $\xi_{\theta_{1}}(x)={\left(\xi_{\theta_{1},1},\cdots ,\xi_{\theta_{1},q}\right)}^{T}$, $\xi_{\theta_{2},u}(x)=\sum_{k=1,k\neq \alpha }^{p}w_{\alpha k}^{-1}\theta_{\alpha k}\xi_{\alpha ku}(x_{\alpha },x_{k})$, $\xi_{\theta_{2}}(x)={\left(\xi_{\theta_{2},1},\cdots ,\xi_{\theta_{2},q}\right)}^{T}$, and $\xi (x)=\xi_{\theta_{1}}(x)+\xi_{\theta_{2}}(x)$. The approximate solution can be represented as a linear combination of basis functions and representers:

(11) $$\hat{g}(x)=\sum_{v=1}^{m}d_{v}\phi_{v}(x)+\sum_{u=1}^{q}c_{u}\left\{\sum_{j=1}^{p}\theta_{j}\xi_{1ju}(x_{j})+\sum_{k=1,k\neq \alpha }^{p}w_{\alpha k}^{-1}\theta_{\alpha ,k}\xi_{\alpha ku}(x_{\alpha },x_{k})\right\}\;\;=\phi^{T}(x)d+\xi^{T}(x)c,$$

where $c={\left(c_{1},\cdots ,c_{q}\right)}^{T}$ and $d={\left(d_{1},\cdots ,d_{m}\right)}^{T}$ are coefficients. Let $Q=\sum_{j=1}^{p}\theta_{j}Q_{j}+\sum_{k=1,k\neq \alpha }^{p}w_{\alpha k}^{-1}\theta_{\alpha k}Q_{\alpha k}$, where $Q_{j}={\left\{R_{j}^{1}({\tilde{x}}_{u,j},{\tilde{x}}_{v,j})\right\}}_{u,v=1}^{q}$ are kernel matrices for the main effects and $Q_{\alpha k}={\left\{R_{\alpha k}(({\tilde{x}}_{u,\alpha },{\tilde{x}}_{u,k}),({\tilde{x}}_{v,\alpha },{\tilde{x}}_{v,k}))\right\}}_{u,v=1}^{q}$ are kernel matrices for the two-way interactions. We can rewrite (10) in a vector form:

(12) $$A(c,d,\theta_{2})=\log\left\{\frac{1}{n}\sum_{i=1}^{n}e^{-\phi_{i}^{T}d-\xi_{i}^{T}c}\right\}+b_{\phi }^{T}d+b_{\xi }^{T}c+\frac{\lambda_{1}}{2}c^{T}Qc+\lambda_{2}w^{T}\theta_{2},$$

where $\phi_{i}=\phi (x_{i})$, $\xi_{i}=\xi (x_{i})$, $b_{\phi }=n^{-1}\sum_{i=1}^{n}\int_{{𝒳}_{\alpha }}\phi (x_{i}^{\alpha })\rho (x_{i}^{\alpha })dx_{\alpha }$, and $b_{\xi }=n^{-1}\sum_{i=1}^{n}\int_{{𝒳}_{\alpha }}\xi (x_{i}^{\alpha })\rho (x_{i}^{\alpha })dx_{\alpha }$. We solve (12) by updating c, d, and $\theta_{2}$ between two steps discussed in the following two subsections.

### 3.1 Newton-Raphson Procedure

We fix $\theta_{2}$ and update c and d at this step. Dropping the last term which is independent of c and d, (12) reduces to

(13) $$A_{1}(c,d)=\log\left\{\frac{1}{n}\sum_{i=1}^{n}e^{-\phi_{i}^{T}d-\xi_{i}^{T}c}\right\}+b_{\phi }^{T}d+b_{\xi }^{T}c+\frac{\lambda_{1}}{2}c^{T}Qc.$$

Note that (13) has the same form as (10.31) in Gu (2013). Therefore, we can solve (13) using the Newton-Raphson procedure with $\lambda_{1}$ and $\theta_{1}$ selected by the approximate cross-validation (ACV) method (Gu, 2013). We note that $\theta_{2}$ are fixed at this step. Therefore, the existing function in the gss R package cannot be used directly. More implementation details can be found in Appendix B.1.

### 3.2 Quadratic Programming

We fix c, d, $\lambda_{1}$ and $\theta_{1}$ and update $\theta_{2}$ at this step. We rewrite $\hat{g}$ in (11) as

(14) $$\hat{g}(x)=\sum_{v=1}^{m}d_{v}\phi_{v}(x)+\sum_{j=1}^{p}\theta_{j}\sum_{u=1}^{q}c_{u}\xi_{1ju}(x_{j})+\sum_{k=1,k\neq \alpha }^{p}\theta_{\alpha k}w_{\alpha k}^{-1}\sum_{u=1}^{q}c_{u}\xi_{\alpha ku}(x_{\alpha },x_{k})\;\;=\phi^{T}(x)d+\psi_{1}^{T}(x)\theta_{1}+\psi_{2}^{T}(x)\theta_{2}.$$

Let $Q_{\left(2\right)}=\sum_{k=1,k\neq \alpha }^{p}w_{\alpha k}^{-1}\theta_{\alpha k}Q_{\alpha k}$. Plugging $\hat{g}(x_{i})$ and keeping terms involving $\theta_{2}$ only, (12) reduces to

(15) $$\log\left\{\frac{1}{n}\sum_{i=1}^{n}e^{-\phi_{i}^{T}d-\psi_{1i}^{T}\theta_{1}-\psi_{2i}^{T}\theta_{2}}\right\}+b_{\psi_{2}}^{T}\theta_{2}+\frac{\lambda_{1}}{2}c^{T}Q_{\left(2\right)}c+\lambda_{2}w^{T}\theta_{2}$$

subject to $\theta_{2}\geq 0$, where $\psi_{1i}=\psi_{1}(x_{i})$, $\psi_{2i}=\psi_{2}(x_{i})$, and $b_{\psi_{2}}=\frac{1}{n}\sum_{i=1}^{n}\int_{{𝒳}_{\alpha }}\psi_{2}(x_{i}^{\alpha })\rho (x_{i}^{\alpha })dx_{\alpha }$. Furthermore, the constraint minimization problem (15) is equivalent to

(16) $$A_{2}(\theta_{2})=\log\left\{\frac{1}{n}\sum_{i=1}^{n}e^{-\phi_{i}^{T}d-\psi_{1i}^{T}\theta_{1}-\psi_{2i}^{T}\theta_{2}}\right\}+b_{\psi_{2}}^{T}\theta_{2}+\frac{\lambda_{1}}{2}c^{T}Q_{\left(2\right)}c$$

subject to $\theta_{2}\geq 0$ and $w^{T}\theta_{2}\leq M$ for some constant M, where M controls the sparsity in $\theta_{2}$. We note that $A_{2}(\theta_{2})$ is a convex function of $\theta_{2}$ (see Appendix C for a brief proof). We solve (16) iteratively using quadratic programming. We apply K-fold cross-validation or BIC method to select M. Implementation details can be found in Appendix B.2.

### 3.3 Algorithm

We summarize the whole algorithm as follows. A parameter with superscript (t) denotes its value at the tth iteration.

**Table:** 

Algorithm 1
$$\begin{array}{cc} \; & \;\mathrm{Input}:\;\text{Data frame}\;X\;\text{containing}\;n\;\text{observations with}\;p\;\text{dimensions}.\\ \; & \;\mathrm{Output}:\;\text{Estimated}\;c,\;d,\;\theta_{2},\;\text{and the neighborhood set}\;nb_{G}(\alpha ).\\ \; & \;\;1:\mathrm{Initialization}\;\theta_{2}^{\left(1\right)}=\theta_{2,0},\theta_{2}^{\left(0\right)}=0,\;\text{and}\;t=1.\\ \; & \;\;2:\mathrm{while}\;{\left\|\theta_{2}^{\left(t\right)}-\theta_{2}^{\left(t-1\right)}\right\|}_{2}∕({\left\|\theta_{2}^{\left(t-1\right)}\right\|}_{2}+10^{-6})\geq \varepsilon \;\text{or}\;t=1\;\mathrm{do}:\\ \; & \;\;3:\;\;\text{Fix}\;\theta_{2}^{\left(t\right)},\\ \; & \;\;\;\;\;\;\;\;\;\;\;\;\;\;\;\;\;\;\;\;\;\;\;\;\;\;\;\;\;\;\;\;c^{\left(t\right)},d^{\left(t\right)}\leftarrow \underset{c,d}{\text{argmin}}\;A_{1}(c,d)\\ \; & \;\;\;\;\;\text{with tuning parameters}\;\lambda_{1}^{\left(t\right)}\;\text{and}\;\theta_{1}^{\left(t\right)}\;\text{selected by the ACV method}.\\ \; & \;\;4:\;\;\text{Fix}\;d^{\left(t\right)},c^{\left(t\right)},\lambda_{1}^{\left(t\right)},\;\text{and}\;\theta_{1}^{\left(t\right)},\\ \; & \;\;\;\;\;\;\;\;\;\;\;\;\;\;\;\;\;\;\;\;\;\;\;\;\;\;\;\;\;\;\;\;\theta_{2}^{\left(t+1\right)}\leftarrow \underset{\theta_{2}}{\text{argmin}}\;A_{2}(\theta_{2}),\\ \; & \;\;\;\;\;\text{subject to}\;\theta_{2}\geq 0\;\text{and}\;w^{T}\theta_{2}\leq M^{\left(t\right)}\;\text{where the tuning parameter}\;M^{\left(t\right)}\;\text{is selected by}\\ \; & \;\;\;\;\;K\text{-fold cross-validation or BIC method}.\\ \; & \;\;5:\;\;t\leftarrow t+1\\ \; & \;\;6:\mathrm{end}\;\mathrm{while} \end{array}$$

More implementation details can be found in Appendix B, including the initialization of $\theta_{2}$, the convergence criterion, and the selection of M.

## 4. Theoretical Analysis

In this section, we study the theoretical properties of the proposed method. Following similar steps and under the same regularity conditions as Gu (2013), we derive the convergence rate for the conditional density estimate $\hat{g}$ subject to both $L_{1}$ and $L_{2}$ penalties. In addition, we derive the convergence rate for interactions in the SS ANOVA decomposition, which is new and important for edge detection.

Let $f_{0}(x_{\alpha }|x_{\setminus \{\alpha \}})\;=\;e^{g_{0}(x)}\rho (x)$ be the true conditional density to be estimated. Let $g\;=\;g^{\left(1\right)}+g^{\left(2\right)}$ where $g^{\left(1\right)}\;=\;\sum_{j=1}^{p}\eta_{j}$ and $g^{\left(2\right)}\;=\;\sum_{k\neq \alpha }\eta_{\alpha k}$ are main effects and interactions respectively. Denote $\hat{g}$ as the minimizer of (9). Define

$$V^{*}(h_{1},h_{2})=\int_{{𝒳}_{\setminus \{\alpha \}}}f_{\setminus \{\alpha \}}(x_{\setminus \{\alpha \}})\int_{{𝒳}_{\alpha }}h_{1}(x)h_{2}(x)\rho (x)dx_{\alpha }dx_{\setminus \{\alpha \}},\;J_{1}(h_{1},h_{2})=\sum_{j=1}^{p}\theta_{j}^{-1}\int_{{𝒳}_{j}}(P_{j}h_{1})(P_{j}h_{2})dx_{j},\;J_{2}(h_{1},h_{2})=\sum_{k\neq \alpha }w_{\alpha k}{\left(\int_{{𝒳}_{\alpha }}\int_{{𝒳}_{k}}|h_{1,\alpha k}h_{2,\alpha k}|dx_{\alpha }dx_{k}\right)}^{1∕2},\;J_{2}^{*}(h_{1},h_{2})=\sum_{k\neq \alpha }\theta_{\alpha k}^{-1}\int_{{𝒳}_{\alpha }}\int_{{𝒳}_{k}}h_{1,\alpha k}h_{2,\alpha k}dx_{\alpha }dx_{k},$$

for any functions $h_{1}$, $h_{2}\;\in \;𝒢$, where $f_{\setminus \{\alpha \}}(x_{\setminus \{\alpha \}})$ is the density of $X_{\setminus \{\alpha \}}$ on ${𝒳}_{\setminus \{\alpha \}}={𝒳}_{1}\times \cdots \times {𝒳}_{\alpha -1}\times {𝒳}_{\alpha +1}\times \cdots \times {𝒳}_{p}$. Furthermore, we define $V^{*}(g)=V^{*}(g,g)$, $V_{1}(g^{\left(1\right)})=V^{*}(g^{\left(1\right)})$, $V_{2}(g^{\left(2\right)})={\left[V^{*}(g^{\left(2\right)})\right]}^{1∕2}$, $J_{1}(g^{\left(1\right)})=J_{1}(g^{\left(1\right)},g^{\left(1\right)})=\sum_{j=1}^{p}\theta_{j}^{-1}{\left\|P_{j}\eta_{j}\right\|}^{2}$, $J_{2}(g^{\left(2\right)})=J_{2}(g^{\left(2\right)},g^{\left(2\right)})=\sum_{k\neq \alpha }w_{\alpha k}\|\eta_{\alpha k}\|$, and $J_{2}^{*}(g^{\left(2\right)})=J_{2}^{*}(g^{\left(2\right)},g^{\left(2\right)})=\sum_{k\neq \alpha }\theta_{\alpha k}^{-1}{\left\|\eta_{\alpha k}\right\|}^{2}$.

Without loss of generality, we assume $w_{\alpha k}=1$ in the proof, simulations, and real data examples. We note that $V^{*}$, $J_{1}$, and $J_{2}^{*}$ are quadratic functionals. In the proof of Corollary 1 in Appendix C, it is shown that $V^{*}(g)$, $J_{1}(g^{\left(1\right)})$, and $J_{2}^{*}(g^{\left(2\right)})$ are equivalent to ${\left\|g\right\|}_{2}^{2}$, $\sum_{j=1}^{p}{\left\|P_{j}\eta_{j}\right\|}_{2}^{2}$, and $\sum_{k\neq \alpha }^{p}{\left\|\eta_{\alpha k}\right\|}_{2}^{2}$, respectively, where ${\left\|\cdot \right\|}_{2}$ is the $L_{2}$ norm. It is also shown that $V_{2}(g^{\left(2\right)})$ and $J_{2}(g^{\left(2\right)})$ are equivalent to the square root of $V^{*}(g^{\left(2\right)})$ and $J_{2}^{*}(g^{\left(2\right)})$. Let $V(g)=V_{1}(g^{\left(1\right)})+V_{2}(g^{\left(2\right)})$, $J=J_{1}+J_{2}$, and $J^{*}(g)=J_{1}(g)+J_{2}^{*}(g)$. To derive the convergence rate, we need the following conditions.

**Condition 1** $V^{*}$ *is completely continuous with respect to* $J^{*}$.

From Theorem 3.1 of Weinberger (1974), there exist eigenvalues $\gamma_{v}$ of $J^{*}$ with respect to $V^{*}$ and the associated eigenfunctions $\zeta_{v}$ such that $V^{*}(\zeta_{v},\zeta_{u})=\delta_{v,u}$ and $J^{*}(\zeta_{v},\zeta_{u})=\gamma_{v}\delta_{v,u}$, where $0\leq \gamma_{v}↑\infty$ and $\delta_{v,u}$ is the Kronecker delta. Functions satisfying $J^{*}(g)<\infty$ can be expressed as a Fourier series expansion $g=\sum_{v}a_{v}\zeta_{v}$, where $a_{v}=V^{*}(g,\zeta_{v})$ are the Fourier coefficients.

**Condition 2** *For* v *sufficiently large and some* φ>0*, the eigenvalues* $\gamma_{v}$ *of* $J^{*}$ *with respect to* $V^{*}$ *satisfy* $\gamma_{v}>\varphi v^{r}$ *where* r>1.

Consider the quadratic functional

(17) $$\frac{1}{n}\sum_{i=1}^{n}-e^{-g_{0}(X_{i})}g(X_{i})+\frac{1}{n}\sum_{i=1}^{n}\int_{{𝒳}_{\alpha }}g(x_{i}^{\alpha })\rho (x_{i}^{\alpha })dx_{\alpha }+\frac{1}{2}V^{*}(g-g_{0})+\frac{\lambda_{1}}{2}J^{*}(g),$$

and denote the minimizer of (17) as $\tilde{g}$. Plugging the Fourier series expansions $g=\sum_{v}a_{v}\zeta_{v}$ and $g_{0}=\sum_{v}a_{v,0}\zeta_{v}$ into (17), $\tilde{g}$ has Fourier coefficients ${\tilde{a}}_{v}=\left(\kappa_{v}+a_{v,0}\right)∕(1+\lambda_{1}\gamma_{v})$, where $\kappa_{v}=n^{-1}\sum_{i=1}^{n}\{e^{-g_{0}(X_{i})}\zeta_{v}(X_{i})-\int_{{𝒳}_{\alpha }}\zeta_{v}(x)\rho (x)dx_{\alpha }\}$. It is not difficult to verify that $E(\kappa_{v})=0$ and $E(\kappa_{v}^{2})\leq n^{-1}\int_{{𝒳}_{\setminus \{\alpha \}}}f_{\setminus \{\alpha \}}(x_{\setminus \{\alpha \}})\int_{{𝒳}_{\alpha }}\zeta_{v}^{2}(x)e^{-g_{0}(x)}\rho (x)dx_{\alpha }dx_{\setminus \{\alpha \}}$.

**Condition 3** *For some* $c_{1}<\infty$, $e^{-g_{0}}<c_{1}$.

Under Condition 3, noting that $V^{*}(\zeta_{v})=\int_{{𝒳}_{\setminus \{\alpha \}}}f_{\setminus \{\alpha \}}(x_{\setminus \{\alpha \}})\int_{{𝒳}_{\alpha }}\zeta_{v}^{2}(x)\rho (x)dx_{\alpha }dx_{\setminus \{\alpha \}}=1$ by the definition of $V^{*}$ and $\zeta_{v}$, we have $E(\kappa_{v}^{2})\leq n^{-1}c_{1}$.

**Condition 4** *For* g *in a convex set* $B_{0}$ *around* $g_{0}$ *containing* $\hat{g}$ *and* $\tilde{g}$, $c_{2}<e^{g_{0}-g}<c_{3}$ *holds uniformly for some* $0<c_{2}<c_{3}<\infty$.

**Condition 5** *For any* u,v=1,2,⋯, $\int_{{𝒳}_{\setminus \{\alpha \}}}f_{\setminus \{\alpha \}}(x_{\setminus \{\alpha \}})\int_{{𝒳}_{\alpha }}\zeta_{v}^{2}\zeta_{u}^{2}e^{-g_{0}}\rho (x)dx_{\alpha }dx_{\setminus \{\alpha \}}<c_{4}$ *for some* $c_{4}<\infty$.

Conditions 1-5 are common assumptions for convergence rate analysis of the SS ANOVA estimates, which were also made in Gu (2013). Condition 2 states that the growth rate of the eigenvalues $\gamma_{v}$ is at $v^{r}$, which controls how fast $\lambda_{1}$ approaches zero. Many commonly used smoothing spline models, including tensor products of cubic splines, thin-plate splines, and spherical splines, satisfy Conditions 1 and 2. See Chapter 9 in Gu (2013) for examples. Condition 4 bounds $e^{g_{0}-g}$ at g in a convex set $B_{0}$ around $g_{0}$. Condition 5 requires a bounded fourth moment of $\zeta_{v}$.

We consider metrics $V^{*}+\lambda_{1}J^{*}$ and $V+\lambda_{1}J$. Let Y>0, we denote $X=O_{p}(Y)$ if P(∣X∣>CY)→0 for some constant C<∞, and denote $X=o_{p}(Y)$ if P(∣X∣>∊Y)→0 for ∀∊>0.

**Theorem 1** *Assume* $\sum_{v}\gamma_{v}^{l}a_{v,0}^{2}<\infty$ *for some* l∈[1,2]. *Under* Conditions 1-5*, for some* r>1, *as* $\lambda_{1}\to 0$ *and* $n\lambda_{1}^{2∕r}\to \infty$,

$$(V^{*}+\lambda_{1}J^{*})(\hat{g}-g_{0})=O_{p}(n^{-1}\lambda_{1}^{-1∕r}+\lambda_{1}^{l}).$$

**Theorem 2** *Under the conditions in Theorem 1,*

$$(V+\lambda_{1}J)(\hat{g}-g_{0})=O_{p}(n^{-1∕2}\lambda_{1}^{-1∕2r}+\lambda_{1}^{l∕2}).$$

**Corollary 1** *Assume conditions in Theorem 2 hold,* $0<c_{5}<\rho (x)<c_{6}$ *and* $0<c_{7}<f_{\setminus \{\alpha \}}(x_{\setminus \{\alpha \}})<c_{8}$ *for some positive constants* $c_{5}$, $c_{6}$, $c_{7}$*, and* $c_{8}$*, we have*

$${\left\|{\hat{\eta }}_{\alpha k}-\eta_{0\alpha k}\right\|}_{2}=O_{p}(n^{-1∕2}\lambda_{1}^{-1∕2r}+\lambda_{1}^{l∕2}),\;\;k\neq \alpha ,\;k=1,\cdots ,p,$$

*where* $\eta_{0\alpha k}$ *are two-way interactions in the true function* $g_{0}$.

We note that $V+\lambda_{1}J$ and $V^{*}+\lambda_{1}J^{*}$ are associated with the $L_{2}$ norm and its square, respectively. Consequently, the convergence rate in Theorem 2 is the square root of the rate in Theorem 1. Corollary 1 holds because $V_{2}$ and $J_{2}$ associated with two-way interactions are equivalent to the $L_{2}$ norm. Consequently, two-way interactions under the $L_{2}$ norm have the same convergence rate as that in Theorem 2. We only show the convergence rate for interactions in Corollary 1 since we are mainly interested in edge selection. Proofs of all theoretical results are in Appendix C.

## 5. Simulation Results

We conduct simulations to evaluate the performance of the proposed method and compare it with some existing methods. We consider four scenarios: multivariate Gaussian, multivariate skewed Gaussian, a directed acyclic graph, and a Gaussian-Bernoulli mixed graphical model.

In implementing the proposed method, we estimate the conditional density for each continuous variable on the data range and transform the data into [0, 1]. We construct an SS ANOVA model using the tensor product of cubic spline models. Specifically, let ${ℋ}^{\left(j\right)}=W_{2}^{2}[0,1]$ where

(18) $$W_{2}^{2}[0,1]=\left\{f\;:f,f^{\prime }\;\text{are absolutely continuous},\int_{0}^{1}{\left(f^{\prime \prime }\right)}^{2}dx<\infty \right\}$$

is the Sobolev space for cubic spline models. Each ${ℋ}^{\left(j\right)}$ can be decomposed as ${ℋ}^{\left(j\right)}=\{1_{\left(j\right)}\}⊕{ℋ}_{\left(j\right)}$ and ${ℋ}_{\left(j\right)}={ℋ}_{\left(j\right)}^{0}⊕{ℋ}_{\left(j\right)}^{1}$, where ${ℋ}_{\left(j\right)}^{0}$ and ${ℋ}_{\left(j\right)}^{1}$ are RKHS’s with reproducing kernels $R_{j}^{0}(x,z)=k_{1}(x)k_{1}(z)$ and $R_{j}^{1}(x,z)=k_{2}(x)k_{2}(z)-k_{4}(|x-z|)$ respectively, $k_{1}(x)=x-0.5$, $k_{2}(x)=\frac{1}{2}(k_{1}^{2}(x)-\frac{1}{12})$, and $k_{4}(x)=\frac{1}{24}(k_{1}^{4}(x)-\frac{k_{1}^{2}(x)}{2}+\frac{7}{240})$. SS ANOVA decomposition of $\overset{p}{\underset{j=1}{⊗}}{ℋ}^{\left(j\right)}$ can then be constructed based on these decompositions. More details can be found in Wang (2011). In all simulations and real data applications, when using the pseudo log-likelihood method, we set

(19) $$\rho (x_{\alpha },x_{\setminus \{\alpha \}})=\frac{\phi ((x_{\alpha }-\mu (x_{\setminus \{\alpha \}}))∕\sigma )}{\Phi (\left(1-\mu (x_{\setminus \{\alpha \}})\right)∕\sigma )-\Phi (\left(-\mu (x_{\setminus \{\alpha \}})\right)∕\sigma )},$$

where ϕ(⋅) and Φ(⋅) are the standard normal density and cumulative distribution functions, and μ(⋅) and σ are estimated by fitting a nonparametric regression model in model space (4) with covariates $x_{\setminus \{\alpha \}}$. More estimation details can be found in Chapter 3 of Gu (2013). We select the tuning parameter M using the 5-fold cross-validation method in all simulations.

For the first three scenarios where all variables are continuous, we compare the proposed method with four existing parametric and semiparametric methods: space (Sparse PArtial Correlation Estimation) (Peng et al., 2009), QUIC (Quadratic Inverse Covariance estimation) (Hsieh et al., 2011), nonparanormal (NPN) (Liu et al., 2009), and SpaCE JAM (Voorman et al., 2014). Due to memory constraints, we will not compare the proposed method with the nonparametric joint density estimation method in Gu et al. (2013).

The space method assumes that E(X)=0 and Cov(X)=Σ. Denote the precision matrix $\Omega =\Sigma^{-1}={\left(\sigma^{ij}\right)}_{p\times p}$ and $\rho^{ij}=-\sigma^{ij}∕\sqrt{\sigma^{ii}\sigma^{jj}}$ as the partial correlation between $X_{i}$ and $X_{j}$. Denote $x_{\left(i\right)}={\left(x_{1,i},\cdots ,x_{n,i}\right)}^{T}$ as the vector of n observations on the ith variable, i=1,⋯,p. Peng et al. (2009) solved the following regularization problem for edge selection

(20) $$\frac{1}{2}\left(\sum_{i=1}^{p}w_{i}{\left\|x_{\left(i\right)}-\sum_{j\neq i}\rho^{ij}\sqrt{\frac{\sigma^{jj}}{\sigma^{ii}}}x_{\left(j\right)}\right\|}^{2}\right)+\lambda \sum_{1\leq i<j\leq p}|\rho^{ij}|,$$

where $w_{i}’s$ are non-negative weights. We implement the space method using the R package space with weights $w_{i}=1$ and tuning parameter λ selected by the 5-fold cross-validation method (Lafit et al., 2019).

The QUIC method assumes that X is multivariate Gaussian and learns the precision matrix Ω by solving the following penalized negative log-likelihood

(21) $$-\log\;\text{det}(\Omega )+\text{tr}(S\Omega )+\lambda {\left\|\Omega \right\|}_{1},$$

where ${\left\|\cdot \right\|}_{1}$ is the $L_{1}$ penalty, S is the sample covariance matrix, and λ is the tuning parameter. We implement the QUIC method using the R package QUIC and select λ using the BIC method.

The NPN method assumes that there exists some monotone functions $f_{1},\cdots ,f_{p}$ such that f(X)∼𝒩(μ,Σ) where $f(X)={\left(f_{1}(X_{1}),\cdots ,f_{p}(X_{p})\right)}^{T}$. The NPN is a semiparametric model since it consists of parameters μ and Σ and nonparametric transformations f’s. The graphical lasso is applied to the transformed data to estimate the undirected graph. Estimation details were given in Liu et al. (2009). We use the R package huge to implement the NPN method with the tuning parameter selected by an extended BIC score (Foygel and Drton, 2010).

The SpaCE JAM method models the conditional mean using additive models: $E(X_{j}|X_{\setminus \{j\}})=\sum_{k\neq j}f_{jk}(X_{k})$ where $f_{jk}(\cdot )$ belongs to a functional space ℱ (Voorman et al., 2014). The functions $f_{jk}$ are estimated as the minimizers of the following least squares with a group lasso type penalty:

(22) $${\text{argmin}}_{f_{jk}\in ℱ}\left\{\frac{1}{2n}\sum_{j=1}^{p}{\left\|x_{\left(j\right)}-\sum_{k\neq j}s_{jk}\right\|}_{2}^{2}+\lambda \sum_{k>j}{\left({\left\|s_{jk}\right\|}_{2}^{2}+{\left\|s_{kj}\right\|}_{2}^{2}\right)}^{1∕2}\right\},$$

where $s_{jk}={\left(f_{jk}(x_{1,k}),\cdots ,f_{jk}(x_{n,k})\right)}^{T}$ and $s_{kj}={\left(f_{kj}(x_{1,j}),\cdots ,f_{kj}(x_{n,j})\right)}^{T}$. We implement the SpaCE JAM method using the R package spacejam (Voorman et al., 2014) with cubic basis functions for non-linear conditional relationships among variables. The tuning parameter λ is selected by the BIC method.

The last scenario comes from Chen et al. (2015), where half of the variables are Gaussian and half are Bernoulli. Chen et al. (2015) assumed a parametric mixed graphical model where each node’s conditional distribution is in the exponential family. Specifically, they considered conditional densities of the form

(23) $$f(x_{\alpha }|x_{\setminus \{\alpha \}})=\text{exp}\left\{h_{\alpha }(x_{\alpha },\beta_{\alpha })+\sum_{k\neq \alpha }\gamma_{\alpha k}x_{\alpha }x_{k}-D_{\alpha }(\varpi_{\alpha }(x_{\setminus \{\alpha \}},\Gamma_{\alpha },\beta_{\alpha }))\right\},$$

where $h_{\alpha }$ is a known function of $x_{\alpha }$ with parameters $\beta_{\alpha }$ and $\varpi_{\alpha }$ is a known function of $x_{\setminus \{\alpha \}}$ with parameters $\Gamma_{\alpha }={\left(\gamma_{\alpha 1},\cdots ,\gamma_{\alpha (\alpha -1)},\gamma_{\alpha (\alpha +1)},\cdots ,\gamma_{\alpha p}\right)}^{T}$ and $\beta_{\alpha }$. Chen et al. (2015) selected the neighborhood set by maximizing the following penalized log-likelihoods for each node:

(24) $$\underset{\Gamma_{\alpha },\beta_{\alpha }}{\text{arg}\;\text{min}}-l_{\alpha }(\Gamma_{\alpha },\beta_{\alpha };X)+\lambda {\left\|\Gamma_{\alpha }\right\|}_{1},$$

where $l_{\alpha }$ is the log-likelihood function. We refer to the method in Chen et al. (2015) as the CEF (Conditional Exponential Family) method. We implement the CEF method using author’s R codes deposited at https://github.com/ChenShizhe/MixedGraphicalModels. We select the tuning parameter λ using the BIC method.

We note that space and CEF are neighborhood selection methods while QUIC, NPN, and SpaCE JAM are global methods. To evaluate the performance of edge detection, we compute three criteria: specificity (SPE), sensitivity (SEN), and $F_{1}$ scores, which are defined as follows:

$$\text{SPE}=\frac{\text{TN}}{\text{TN}+\text{FP}},\;\;\;\text{SEN}=\frac{\text{TP}}{\text{TP}+\text{FN}},\;\;\;F_{1}=\frac{2\text{TP}}{2\text{TP}+\text{FN}+\text{FP}},$$

where TP, TN, FP, and FN are the numbers of true positives, true negatives, false positives and false negatives.

We set dimension p=20 and consider two sample sizes n=150 and n=300. All simulations are repeated for 100 times.

### 5.1 Multivariate Gaussian

In this section, we generate data from Gaussian distributions with different precision matrices. We first use huge.generator function to randomly generate a p×p sparse precision matrix Ω, where the probability $p_{\text{off}}$ of the off-diagonal elements being nonzero is equal to 0.2 or 0.4. Then, we generate n i.i.d. samples $X_{1},\cdots ,X_{n}$ from $𝒩(0,\Omega^{-1})$. We apply the proposed method and compare its performance with the space, QUIC, NPN, and SpaCE JAM methods.

Table 1 presents averages and standard deviations of the sensitivity, specificity, and $F_{1}$ score. In general, the performances of all methods are better for the larger sample size. In most settings, all methods perform better when the precision matrix is sparser (i.e. $p_{\text{off}}=0.2$). Different methods have different trade-offs between sensitivity and specificity. Overall, the NPN method has inferior performance compared to other methods, which is expected since the true distribution is Gaussian. In general, the SpaCE JAM performs better than QUIC in specificity and $F_{1}$ score. This result agrees with the observations of Voorman et al. (2014) that SpaCE JAM tends to outperform the NPN and graphical lasso methods. The space method has a similar performance as the SpaCE JAM. Unexpectedly, even in this Gaussian case, the proposed method has larger sensitivities and $F_{1}$ scores and reasonable specificities compared to other methods. Therefore, the proposed method is efficient in edge detection and performs better with a more balanced trade-off between specificity and sensitivity even under this multivariate Gaussian scenario.

### 5.2 Multivariate Skewed Gaussian

In this section, we consider the scenario when X follows a multivariate skewed Gaussian distribution with density function (Azzalini and Valle, 1996)

(25) $$f(x)=2\phi_{p}(x;\mu ,\Sigma )\Phi (a^{T}x),$$

where $\phi_{p}(x;\mu ,\Sigma )$ is the p-dimensional normal density with mean μ and covariance matrix Σ, Φ(⋅) is the cumulative distribution function of the standard Gaussian distribution, and a is a p-dimensional vector that controls the skewness of the multivariate Gaussian distribution. When a=0, the distribution reduces to the multivariate Gaussian distribution. We set a=a1 and consider two choices of a: a=1 and a=4, where 1 is a p-dimensional vector of all ones. We let μ=0.51 and randomly generate $\Sigma^{-1}$ as a p×p matrix, where the probability of the off-diagonal elements being nonzero equals 0.4. True edges correspond to nonzero off-diagonal elements of the precision matrix $\Sigma^{-1}$.

Table 2 presents averages and standard deviations of sensitivity, specificity, and $F_{1}$ score. All methods have better performances under the larger sample size. Again, different methods have different trade-offs between sensitivity and specificity. The space, NPN, and SpaCE JAM methods have small sensitivities and $F_{1}$ scores when n=150. As expected, the proposed method has the best overall performance with significantly larger sensitivity and $F_{1}$ score and reasonable specificity.

### 5.3 Directed Acyclic Graph

It is generally difficult to construct a flexible multivariate nonparametric distribution, as discussed in Section 2 in Voorman et al. (2014). To overcome this problem, we use the same approach in Voorman et al. (2014) to generate a graphical model using a directed acyclic graph (DAG) and conditional distributions. We use the rdag function in the spacejam package to create a DAG of X and denote $E_{D}$ as the directed edge set. The conditional relationships among variables can be created via $E(X_{j}|X_{\setminus \{j\}})=\sum_{k\neq j}f_{jk}(X_{k})$. The distribution of X is usually not a well-known multivariate distribution except for the particular case when all $f_{jk}s$ are linear for multivariate Gaussian distribution.

We decompose $X^{T}=(Y^{T},Z^{T})$ where Y and Z are random vectors of dimensions 5 and 15 respectively. We first generate a DAG with p=20 nodes and m edges selected at random from all possible p(p−1)∕2 possible edges. We consider two choices of m: m=20 and m=40. Given a DAG, we generate data as follows:

$$Z_{j}|\{Z_{k},Y_{s}\;:\{k,j\},\{s,j\}\in E_{D}\}=\sum_{\{k,j\}\in E_{D}}f_{jk}^{\left(1\right)}(Z_{k})+\sum_{\{s,j\}\in E_{D}}f_{js}^{\left(1\right)}(Y_{s})+{∊}_{j}\;\;Y_{j}|\{Y_{k}\;:\{k,j\}\in E_{D}\}=\sum_{\{k,j\}\in E_{D}}f_{jk}^{\left(2\right)}(Y_{k})+{∊}_{j},$$

where ${∊}_{j}’s$ are i.i.d. random noises from the standard normal distribution, $f_{jk}^{\left(1\right)}(t)=b_{jk,1}^{\left(1\right)}t$ with $b_{jk,1}^{\left(1\right)}$ generated from the standard Gaussian distribution, and $f_{jk}^{\left(2\right)}(t)=b_{jk,1}^{\left(2\right)}t+b_{jk,2}^{\left(2\right)}t^{2}+b_{jk,3}^{\left(2\right)}t^{2}$ with $b_{jk,1}^{\left(2\right)}$, $b_{jk,2}^{\left(2\right)}$ and $b_{jk,3}^{\left(2\right)}$ independently generated from the Gaussian distributions with mean zero and variances 1, 0.3, and 0.1, respectively.

Simulation results are shown in Table 3. The performances of all methods are better when the sample size is larger. Different methods have different trade-offs between sensitivity and specificity. Since data are generated according to a model assumed by the SpaCE JAM method, as expected, the SpaCE JAM method performs better in $F_{1}$ score than the space, QUIC, and NPN methods. Remarkably, in all cases, the proposed method has larger $F_{1}$ scores than all methods, including the SpaCE JAM. It is interesting to note that the denser graph (i.e. m=40) reduces the sensitivity of the proposed method and the specificity of the SpaCE JAM method.

### 5.4 Gaussian-Bernoulli Mixed Graphical Model

In this section, we consider a mixed graphical model used in Section 6.1 of Chen et al. (2015). The graph used to generate the data is shown in Figure 1 of Chen et al. (2015). Specifically, there are m Gaussian nodes labeled as 1,⋯,m and m Bernoulli nodes labeled as m+1,⋯,2m. For j=1,⋯,m, the jth and (j+m)th node are connected to its adjacent nodes of the same type, and the jth node and the (j+m)th node are connected to each other. Consider the following model

(26) $$f(x)\propto \text{exp}\left\{\sum_{j=1}^{p}h_{j}(x_{j})+\frac{1}{2}\sum_{k=1}^{p}\sum_{j\neq k}\gamma_{jk}x_{j}x_{k}\right\},$$

where $h_{j}$ is the node potential and $\gamma_{jk}$ are edge potentials. The edge potentials $\gamma_{jk}$ and $\gamma_{kj}$ are generated as

$$\gamma_{jk}=\gamma_{kj}=y_{jk}r_{jk},\;\;P(y_{jk}=1)=P(y_{jk}=-1)=0.5,\;\;r_{jk}∼\text{Unif}(0.3,0.6),$$

and $\gamma_{jk}=\gamma_{kj}=0$ if (j,k)∉E. Gibbs sampling is employed to sample data from (26). In this simulation scenario, we compare the proposed method with the CEF method only since it performed better than other existing methods for mixed data (Chen et al., 2015).

Table 4 shows that the proposed and the CEF methods have different trade-offs between sensitivity and specificity. The proposed method has better sensitivity, while the CEF method has better specificity. The proposed method has slightly better $F_{1}$ scores.

## 6. Applications

We illustrate our neighborhood selection method using two real datasets. Section 6.1 applies our method to Arabidopsis Thaliana gene expression data and compares the estimated graph with those from space, QUIC, NPN, and SpaCE JAM. In addition, we present a diagnostic procedure for some existing methods. Section 6.2 illustrates our method using a dataset with mixed data types.

### 6.1 Isoprenoid Gene Network in Arabidopsis Thaliana

In this section, we consider the gene expression data for *Arabidopsis thaliana,* an important plant species in molecular biology and genetics studies. There are n=118 observations of Affymetrix GeneChip microarrays in the dataset, where a subset of p=39 genes from the isoprenoid pathway is selected for analysis. The dataset was introduced in Wille et al. (2004) and was downloaded at https://www.ncbi.nlm.nih.gov/pmc/articles/PMC545783/. Lafferty et al. (2012) analyzed this dataset using the nonparanormal method.

All observations are preprocessed by log-transformation and standardization as in Lafferty et al. (2012). Using the proposed method, we build a graph for all 39 gene expression levels and compare its structure with those from space, QUIC, NPN, and SpaCE JAM. Wille et al. (2004) stated that the Gaussian graphical model selection with the BIC choice of the tuning parameter usually detects too many edges for biologically-relevant analysis. Therefore, we limit the number of edges in the graph by controlling the regularization parameters as in Lafferty et al. (2012). Specifically, we tune M such that the number of edges ∣E∣=52. Similarly, by tuning the regularization parameters in space, QUIC, NPN, and SpaCE JAM, we select the graphs with the same number of edges ∣E∣=52.

**Figure 1:**
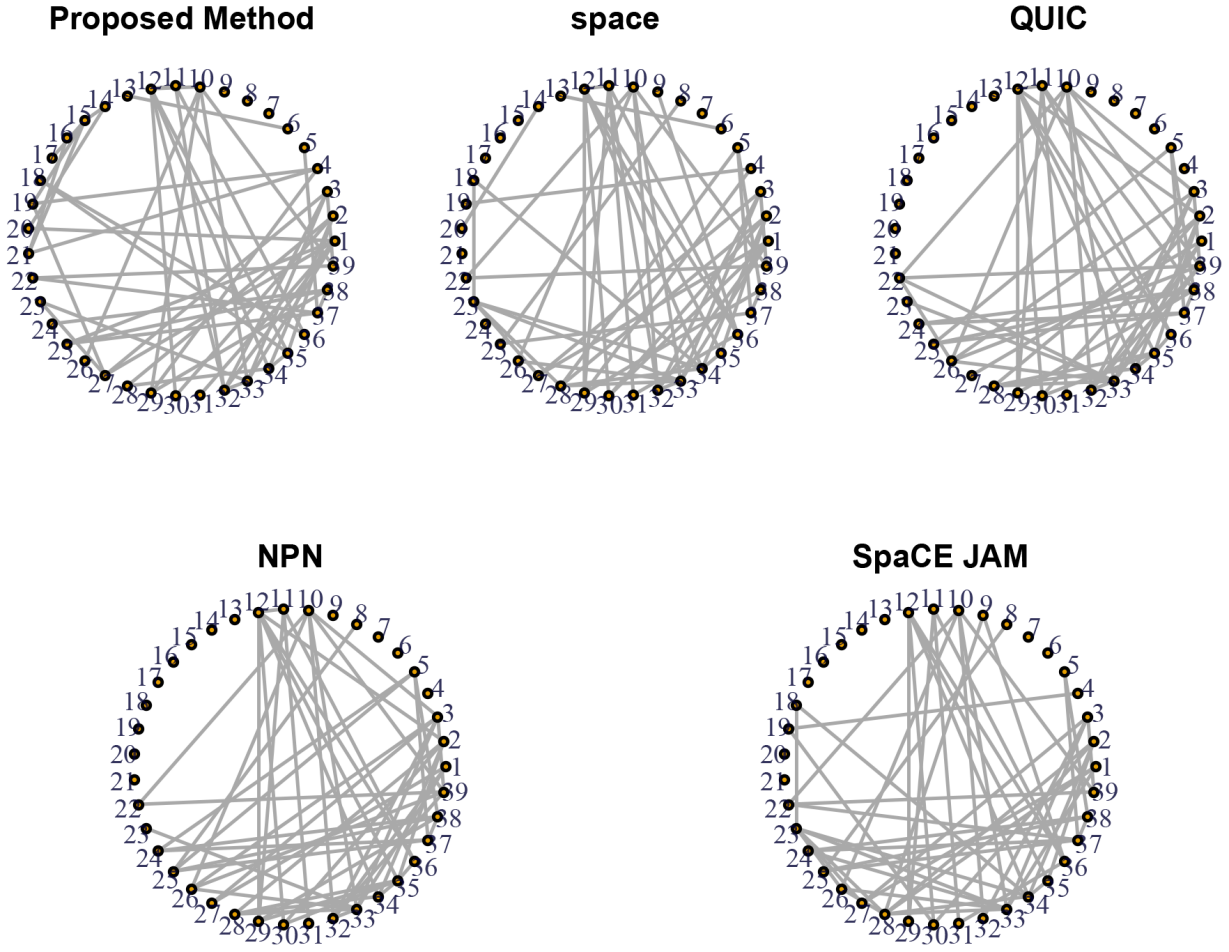
The estimated graph with 52 edges from the proposed (*top left*), the space (*top middle*), the QUIC (*top right*), the NPN (*bottom left*), and the SpaCE JAM (*bottom right*) methods.

Figure 1 presents graphs with ∣E∣=52 for all methods. These five graphs have some common edges, for example, edges 1-27, 1-33, 2-28, 2-30, 2-34, 2-35, 3-32, 3-33, 3-39, 5-37, 10-26, 10-33, 10-39, 11-36, 12-29, 12-30, 12-34, 12-35, 22-39, 23-33, 25-37, 28-34, 34-35, and 37-38. There are also some interesting differences. For instance, only our proposed method detects edge 16-21. We now describe a general diagnostic procedure that explains why other methods miss this edge.

We first extend the squared error projection in Gu (2013) for diagnostics on any subspaces of ${ℳ}_{\alpha }$. Let

(27) $$\tilde{V}(\hat{g}-g)=\int_{{𝒳}_{\setminus \{\alpha \}}}f_{\setminus \{\alpha \}}(x_{\setminus \{\alpha \}})\int_{{𝒳}_{\alpha }}{\left\{(\hat{g}-g)(x)-\int_{{𝒳}_{\alpha }}(\hat{g}-g)(x)\rho (x)\right\}}^{2}\rho (x)dx_{\alpha }dx_{\setminus \{\alpha \}}$$

where $\hat{g}\in {ℳ}_{\alpha }\;⊖\;\{1\}$. We remove the constant functions from the model space since they are not relevant to the diagnostics on interactions. $\tilde{V}(\hat{g}-g)$ can be treated as a proxy of the symmetrized Kullback-Leibler distance (Gu, 2013). For any decomposition ${ℳ}_{\alpha }\;⊖\;\{1\}={ℳ}_{\alpha }^{0}⊕{ℳ}_{\alpha }^{1}$, the squared error projection of $\hat{g}$ in ${ℳ}_{\alpha }^{0}$ is defined as $\tilde{g}=\underset{g\in {ℳ}_{\alpha }^{0}}{\text{arg}\;\text{min}}\left\{\tilde{V}(\hat{g}-g)\right\}$. It can be shown that $\tilde{V}(\hat{g}-g_{u})=\tilde{V}(\hat{g}-\tilde{g})+\tilde{V}(\tilde{g}-g_{u})$ when $g_{u}=-\log\;\rho (x)\in {ℳ}_{\alpha }^{0}$. The ratio $\tilde{V}(\hat{g}-\tilde{g})∕\tilde{V}(\hat{g}-g_{u})$ represents the contribution of functions in subspace ${ℳ}_{\alpha }^{1}$ which can be dropped when the ratio is small (Gu et al., 2013).

Now we apply the diagnostic procedure to explain why our proposed method detects edge 16-21, which is missed by other methods. Note that the interaction space ${ℋ}_{\left(\alpha k\right)}={ℋ}_{\left(\alpha k\right)}^{\left(0\right)}⊕{ℋ}_{\left(\alpha k\right)}^{\left(1\right)}⊕{ℋ}_{\left(\alpha k\right)}^{\left(2\right)}⊕{ℋ}_{\left(\alpha k\right)}^{\left(3\right)}$ where ${ℋ}_{\left(\alpha k\right)}^{\left(0\right)}={ℋ}_{\left(\alpha \right)}^{0}⊗{ℋ}_{\left(k\right)}^{0}$, ${ℋ}_{\left(\alpha k\right)}^{\left(1\right)}={ℋ}_{\left(\alpha \right)}^{0}⊗{ℋ}_{\left(k\right)}^{1}$, ${ℋ}_{\left(\alpha k\right)}^{\left(2\right)}={ℋ}_{\left(\alpha \right)}^{1}⊗{ℋ}_{\left(k\right)}^{0}$, and ${ℋ}_{\left(\alpha k\right)}^{\left(3\right)}={ℋ}_{\left(\alpha \right)}^{1}⊗{ℋ}_{\left(k\right)}^{1}$ correspond to linear-linear, linear-smooth, smooth-linear, and smooth-smooth interactions (Wang, 2011). The QUIC and space are special cases with $\eta_{\alpha k}\in {ℋ}_{\left(\alpha k\right)}^{\left(0\right)}$, and the SpaCE JAM is a special cases with $\eta_{\alpha k}\in {ℋ}_{\left(\alpha k\right)}^{\left(0\right)}⊕{ℋ}_{\left(\alpha k\right)}^{\left(1\right)}$. Therefore, for diagnostics of QUIC and SpaCE JAM methods, we consider the contribution of ${ℳ}_{\alpha }^{1}={ℳ}_{\alpha }\;⊖\;\{1\}\;⊖\;{ℋ}_{\left(\alpha k\right)}^{\left(0\right)}$ and the contribution of ${ℳ}_{\alpha }^{1}={ℳ}_{\alpha }\;⊖\;\{1\}\;⊖\;{ℋ}_{\left(\alpha k\right)}^{\left(0\right)}\;⊖\;{ℋ}_{\left(\alpha k\right)}^{\left(1\right)}$, respectively. For edge 16-21, we have $\tilde{V}\left(\hat{g}-\tilde{g}\right)∕\tilde{V}(\hat{g}-g_{u})=0.352$ for QUIC and $\tilde{V}\left(\hat{g}-\tilde{g}\right)∕\tilde{V}(\hat{g}-g_{u})=0.340$ for SpaCE JAM, respective. These non-ignorable contributions suggest that the assumptions of the QUIC and SpaCE JAM methods are likely violated.

### 6.2 Conditional Dependence Among Demographic, Clinical, Laboratory and Treatment Variables of Hemodialysis Patients

In this section, we illustrate the application of the proposed methods to mixed binary and continuous variables using a data set collected from hemodialysis patients. The data include patients who received dialysis treatments during 2010-2014 and stayed at Fresenius Medical Care - North America throughout their treatments. To reduce heterogeneity, we include n=2932 non-diabetic and non-Hispanic patients who used arteriovenous fistula for dialysis access and survived longer than two years. We use the averages of measurements in the second year of dialysis for analysis. We consider the following 23 variables: demographic variables including race (white and non-white) and gender (male and female); clinical variables including height (cm), weight (kg), sbp (systolic blood pressure, mmHg), dbp (diastolic blood pressure, mmHg), and temp (temperature, Celsius); laboratory variables including albumin (g/dL), ferritin (ng/mL), hgb (hemoglobin, g/dL), lymphocytes (%), neutrophils (%), nlr (neutrophils to lymphocytes ratio, unitless), sna (serum sodium concentration, mEq/L), wbc (white blood cell, 1000/mc); and treatment variables including qb (blood flow, mL/min), qd (dialysis flow, mL/min), saline (mL), olc (on-line clearance, unitless), idwg (interdialytic weight gain, kg), ufv (ultrafiltration volume, L), ufr (ultrafiltration rate, mL/hr/kg), and epodose (erythropoietin dose, unit).

**Figure 2:**
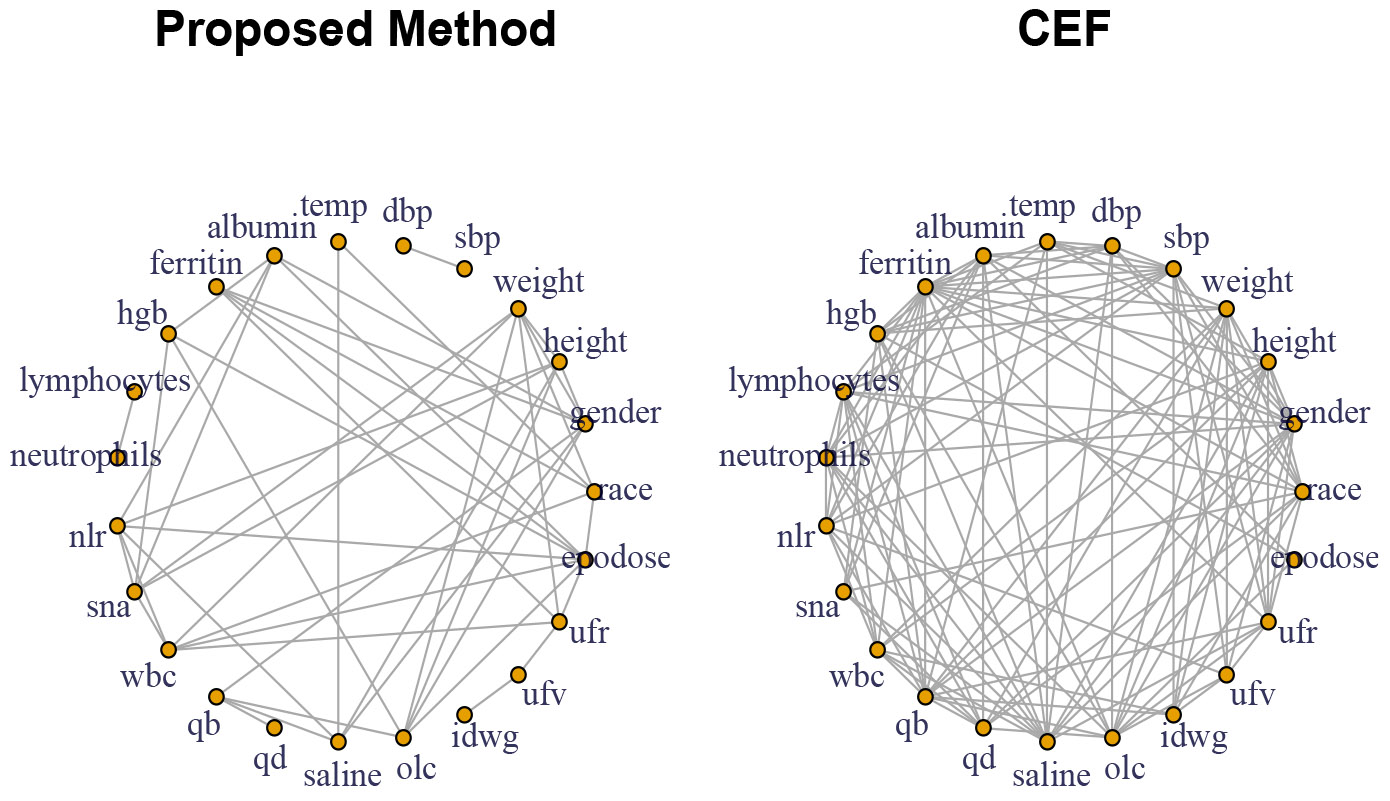
The estimated graph for dialysis data from the proposed (*left*) and the CEF (*right*) methods.

We have 2 binary variables, race and male, and 21 continuous variables. We apply the logistic regression approach described in the Supplement to estimate the conditional density of each binary variable and the pseudo log-likelihood to estimate the conditional density of each continuous variable. We apply the BIC method to select the tuning parameter M. The left panel in Figure 2 shows the estimated graph which contains some of the expected dependencies between variables such as gender and height, weight and height, and sbp and dbp. The link between ufv and idwg is also well-known (Uduagbamen et al., 2021). Many other edges corroborate with existing literature. For example, anemia is a common complication of dialysis patients, and its management is a major challenge. A central aim of anemia management is to maintain patients’ hemoglobin levels consistently within a target range. Erythropoietin has been used to raise hemoglobin levels, which is revealed by the edge between epodose and hgb. Serum albumin has been found to be strongly associated with erythropoietin sensitivity (Agarwal et al., 2008), which is corroborated by the edge between epodose and albumin. It has been found that black patients receive greater doses of erythropoietin than white patients (Lacson et al., 2008), which is corroborated by the edge between epodose and race. The estimated graph from our proposed method in Figure 2 (left panel) provides a holistic view of complex relationships between the demographic, clinical, laboratory, and treatment variables and helps build new theories to be tested in future studies. For comparison, we apply the CEF method to fit the Gaussian-Bernoulli model (26) with the BIC choice of the tuning parameter to this data and show the resulting graph on the right panel in Figure 2. The CEF method leads to a very dense graph.

## 7. Conclusion

This paper develops a fully nonparametric method for neighborhood selection in pairwise graphical models. Since the range of each random variable is an arbitrary set, the proposed method provides a unified framework for mixed data types. The proposed SS ANOVA models are more general than existing parametric and semiparametric models. We develop penalized log-likelihood and pseudo log-likelihood methods with an $L_{1}$ penalty to select edges. As illustrated in Section 6.1, in addition to providing more flexible alternatives, the proposed method also serves as a new diagnostic tool for existing graphical models. We establish convergence rates of the conditional density function estimate and interaction components in the SS ANOVA decomposition. Simulation results show that the proposed method is efficient in edge detection and performs well under Gaussian and non-Gaussian situations. Applications to real data indicate that the proposed method could detect edges that may provide new perspectives for researchers. We note that as a nonparametric method, even though it is parallelizable, the proposed method takes much longer CPU time than parametric and semiparametric methods compared in this paper.

We note that the proposed methods can be easily extended to select variables in non-parametric conditional density estimation, which has not been studied to the best of our knowledge. The proposed method can also be extended to incorporate prior knowledge of the conditional density of a node using a model-based penalty or a semiparametric model (Shi et al., 2019; Yu et al., 2020). For example, it may be known that the conditional density of $X_{\alpha }$ is close to but not necessarily a Gaussian distribution. We may consider a quintic thin-plate spline space for ${ℋ}^{\left(\alpha \right)}$ with a tensor sum decomposition ${ℋ}^{\left(\alpha \right)}={ℋ}_{\left(\alpha \right)}^{0}⊕{ℋ}_{\left(\alpha \right)}^{1}$, where ${ℋ}_{\left(\alpha \right)}^{0}=\{1_{\left(\alpha \right)},x_{\left(\alpha \right)},x_{\left(\alpha \right)}^{2}\}$ corresponds to the space for logistic density of a Gaussian distribution. The edge selection consistency and the control of false positives also warrant further investigation.

## Figures and Tables

**Table 1:** Averages and standard deviations (in parentheses) of specificity (SPE), sensitivity (SEN), and $F_{1}$ score for the multivariate Gaussian scenario.

	Proposed Method			space			QUIC			NPN			SpaCE JAM		
	SPE	SEN	$F_{1}$	SPE	SEN	$F_{1}$	SPE	SEN	$F_{1}$	SPE	SEN	$F_{1}$	SPE	SEN	$F_{1}$
$p_{\text{off}}=0.2$															
n=150	0.912 (0.028)	0.968 (0.037)	0.834 (0.050)	0.986 (0.011)	0.654 (0.096)	0.762 (0.075)	0.768 (0.036)	0.964 (0.034)	0.666 (0.042)	0.820 (0.108)	0.751 (0.402)	0.521 (0.281)	0.939 (0.035)	0.798 (0.147)	0.776 (0.068)
n=300	0.929 (0.026)	0.998 (0.008)	0.870 (0.046)	0.989 (0.01)	0.869 (0.059)	0.907 (0.04)	0.813 (0.032)	0.982 (0.025)	0.712 (0.04)	0.762 (0.042)	0.995 (0.016)	0.668 (0.042)	0.945 (0.022)	0.954 (0.041)	0.875 (0.037)
$p_{\text{off}}=0.4$															
n=150	0.866 (0.040)	0.815 (0.066)	0.807 (0.046)	0.969 (0.013)	0.461 (0.062)	0.599 (0.058)	0.668 (0.047)	0.797 (0.058)	0.691 (0.030)	0.793 (0.142)	0.617 (0.411)	0.474 (0.312)	0.945 (0.025)	0.508 (0.135)	0.608 (0.126)
n=300	0.883 (0.042)	0.968 (0.027)	0.903 (0.028)	0.961 (0.015)	0.564 (0.058)	0.681 (0.049)	0.689 (0.043)	0.827 (0.046)	0.717 (0.026)	0.673 (0.039)	0.951 (0.036)	0.706 (0.021)	0.905 (0.031)	0.704 (0.128)	0.727 (0.076)

**Table 2:** Averages and standard deviations (in parentheses) of specificity (SPE), sensitivity (SEN), and $F_{1}$ score for the multivariate skewed Gaussian scenario.

	Proposed Method			space			QUIC			NPN			SpaCE JAM		
	SPE	SEN	$F_{1}$	SPE	SEN	$F_{1}$	SPE	SEN	$F_{1}$	SPE	SEN	$F_{1}$	SPE	SEN	$F_{1}$
a=1															
n=150	0.892 (0.039)	0.854 (0.058)	0.847 (0.044)	0.957 (0.023)	0.302 (0.053)	0.440 (0.060)	0.688 (0.041)	0.798 (0.055)	0.704 (0.027)	0.873 (0.160)	0.332 (0.412)	0.286 (0.352)	0.926 (0.039)	0.378 (0.160)	0.486 (0.164)
n=300	0.927 (0.034)	0.976 (0.022)	0.937 (0.025)	0.949 (0.027)	0.470 (0.061)	0.608 (0.061)	0.718 (0.045)	0.827 (0.047)	0.737 (0.027)	0.668 (0.049)	0.932 (0.047)	0.769 (0.024)	0.845 (0.088)	0.728 (0.133)	0.739 (0.069)
a=4															
n=150	0.898 (0.034)	0.860 (0.056)	0.854 (0.040)	0.958 (0.023)	0.299 (0.054)	0.438 (0.063)	0.692 (0.040)	0.800 (0.055)	0.707 (0.027)	0.861 (0.164)	0.357 (0.415)	0.306 (0.355)	0.929 (0.037)	0.384 (0.161)	0.493 (0.166)
n=300	0.917 (0.034)	0.979 (0.019)	0.931 (0.026)	0.950 (0.026)	0.473 (0.060)	0.610 (0.059)	0.772 (0.041)	0.828 (0.047)	0.739 (0.027)	0.673 (0.049)	0.934 (0.045)	0.773 (0.023)	0.834 (0.103)	0.748 (0.140)	0.745 (0.067)

**Table 3:** Averages and standard deviations (in parentheses) of specificity (SPE), sensitivity (SEN), and $F_{1}$ score for the directed acyclic graph scenario.

	Proposed Method			space			QUIC			NPN			SpaCE JAM		
	SPE	SEN	$F_{1}$	SPE	SEN	$F_{1}$	SPE	SEN	$F_{1}$	SPE	SEN	$F_{1}$	SPE	SEN	$F_{1}$
m=20															
n=150	0.970 (0.020)	0.835 (0.074)	0.840 (0.066)	0.997 (0.004)	0.588 (0.084)	0.730 (0.068)	0.808 (0.034)	0.838 (0.079)	0.588 (0.050)	0.83 (0.066)	0.791 (0.125)	0.588 (0.054)	0.963 (0.019)	0.697 (0.079)	0.736 (0.062)
n=300	0.984 (0.013)	0.917 (0.064)	0.915 (0.050)	0.998 (0.003)	0.631 (0.075)	0.767 (0.058)	0.859 (0.035)	0.854 (0.079)	0.660 (0.055)	0.818 (0.041)	0.894 (0.082)	0.629 (0.052)	0.979 (0.057)	0.716 (0.089)	0.786 (0.072)
m=40															
n=150	0.970 (0.020)	0.598 (0.064)	0.725 (0.05)	0.984 (0.012)	0.359 (0.039)	0.517 (0.041)	0.705 (0.039)	0.671 (0.053)	0.627 (0.031)	0.671 (0.042)	0.708 (0.060)	0.634 (0.031)	0.690 (0.111)	0.710 (0.112)	0.643 (0.044)
n=300	0.985 (0.014)	0.685 (0.067)	0.800 (0.049)	0.982 (0.013)	0.430 (0.054)	0.587 (0.050)	0.740 (0.046)	0.673 (0.049)	0.645 (0.036)	0.707 (0.045)	0.724 (0.048)	0.662 (0.038)	0.654 (0.07)	0.814 (0.051)	0.692 (0.031)

**Table 4:** Averages and standard deviations (in parentheses) of specificity (SPE), sensitivity (SEN), and $F_{1}$ score for the Gaussian-Bernoulli mixed graphical model.

	Proposed Method			CEF		
	SPE	SEN	$F_{1}$	SPE	SEN	$F_{1}$
n=150	0.897 (0.021)	0.752 (0.053)	0.656 (0.044)	0.947 (0.020)	0.450 (0.015)	0.509 (0.035)
n=300	0.900 (0.022)	0.804 (0.042)	0.675 (0.039)	0.934 (0.021)	0.467 (0.017)	0.504 (0.032)

## Appendix A. Penalized Log-likelihood Estimation

We describe the general penalized log-likelihood approach in Section A.1 and how it is applied for binary variables in Section A.2.

### A.1 Log-likelihood Approach for Conditional Density Estimation

In this section, we describe the log-likelihood approach for estimating conditional density with $L_{1}$ penalty. For identifiability, the constant and main effects of $x_{\setminus \{\alpha \}}$ are removed from the model space. Specifically, the model space for η in (3) is assumed to be

(28)
$${ℳ}_{\alpha }^{*}=\{{ℋ}_{\left(\alpha \right)}\}⊕\left\{\underset{k\neq \alpha }{⊕}[{ℋ}_{\left(\alpha \right)}⊗{ℋ}_{\left(k\right)}]\right\}.$$

A function $\eta \in {ℳ}_{\alpha }^{*}$ can be decomposed as follows:

(29)
$$\eta (x)=\eta_{j}(x_{\alpha })+\sum_{k\neq \alpha }\eta_{\alpha k}(x_{\alpha },x_{k}),$$

where each component in (29) belongs to the corresponding subspace in (28). We further decompose ${ℋ}_{\left(\alpha \right)}$ as ${ℋ}_{\left(\alpha \right)}={ℋ}_{\left(\alpha \right)}^{0}⊕{ℋ}_{\left(\alpha \right)}^{1}$ where ${ℋ}_{\left(\alpha \right)}^{0}$ is a finite-dimensional space containing functions that are not subject to the $L_{2}$ penalty. We estimate η by minimizing the following penalized log-likelihood in ${ℳ}_{\alpha }^{*}$:

(30)
$$l_{\alpha }^{*}+\frac{\lambda_{1}}{2}\left(\theta_{\alpha }^{-1}{\left\|P_{\alpha }\eta_{\alpha }\right\|}^{2}+\sum_{k\neq \alpha }w_{\alpha k}\theta_{\alpha k}^{-1}{\left\|\eta_{\alpha k}\right\|}^{2}\right)+\lambda_{2}\sum_{k\neq \alpha }w_{\alpha k}\theta_{\alpha k},$$

where $l_{\alpha }^{*}=-n^{-1}\sum_{i=1}^{n}\left\{\eta (x_{i})-\log\;\int_{{𝒳}_{\alpha }}e^{\eta (x_{\alpha },x_{i,\setminus \{\alpha \}})}dx_{\alpha }\right\}$, and $P_{\alpha }$ is the projection operator onto ${ℋ}_{\left(\alpha \right)}^{1}$. Let $\theta_{2}\;=\;{\left(\theta_{\alpha 1},\cdots ,\theta_{\alpha (\alpha -1)},\theta_{\alpha (\alpha +1)},\cdots ,\theta_{\alpha p}\right)}^{T}$, $\phi_{\alpha }\;=\;{\left(\phi_{\alpha 1},\cdots ,\phi_{\alpha m_{\alpha }}\right)}^{T}$ be a vector of basis functions of ${ℋ}_{\left(\alpha \right)}^{0}$, $\xi_{u}(x)\;=\;\theta_{\alpha }\xi_{1\alpha u}(x_{\alpha })+\sum_{k=1,k\neq \alpha }^{p}w_{\alpha k}^{-1}\theta_{\alpha ,k}\xi_{\alpha ku}(x_{\alpha },x_{k})$ for u=1,⋯,q, and $\xi (x)={\left(\xi_{1}(x),\cdots ,\xi_{q}(x)\right)}^{T}$.

Similar to (11), the approximate solution can be represented as

(31)
$$\hat{\eta }(x)=\sum_{v=1}^{m_{\alpha }}d_{v}\phi_{\alpha v}(x)+\sum_{u=1}^{q}c_{u}\left\{\theta_{\alpha }\xi_{1\alpha u}(x_{\alpha })+\sum_{k=1,k\neq \alpha }^{p}w_{\alpha k}^{-1}\theta_{\alpha ,k}\xi_{\alpha ku}(x_{\alpha },x_{k})\right\}\;\;=\phi_{\alpha }^{T}(x)d+\xi^{T}(x)c,$$

where $c={\left(c_{1},\cdots ,c_{q}\right)}^{T}$ and $d={\left(d_{1},\cdots ,d_{m_{\alpha }}\right)}^{T}$. Plugging $\hat{\eta }(x_{i})$ in (31) into (30), we need to compute c, d, and $\theta_{2}$ as the minimizers of

(32)
$$-\frac{1}{n}\sum_{i=1}^{n}(\phi_{\alpha ,i}^{T}d+\xi_{i}^{T}c)+\frac{1}{n}\sum_{i=1}^{n}\log\int_{{𝒳}_{\alpha }}e^{\phi_{\alpha }^{T}(x_{\alpha },x_{i,\setminus \{\alpha \}})d+\xi^{T}(x_{\alpha },x_{i,\setminus \{\alpha \}})c}dx_{\alpha }+\frac{\lambda_{1}}{2}c^{T}Qc+\lambda_{2}w^{T}\theta_{2}$$

subject to $\theta_{2}\geq 0$. Similar to the Algorithm in Section 3, we can update c, d, and $\theta_{2}$ sequentially. The estimate of $\theta_{2}$ provides the selection results. We skip the details here because the derivation is similar to Section 3. The algorithm can be similarly implemented as described in Appendix B. With minor changes in Conditions 1-5 and the definition of V, following similar steps in Appendix C, it can be shown that Theorem 1, Theorem 2, and Corollary 1 hold for the log-likelihood approach with the same convergence rates.

### A.2 Logistic Regression for Binary Variables

In this section, we consider the penalized log-likelihood approach for the special case when $x_{\alpha }$ is a binary variable taking values 0 or 1. Consider the logit function

$$\nu (x)\;=\;\log\{f(1|x_{\setminus \{\alpha \}})∕f(0|x_{\setminus \{\alpha \}})\}\;\;=\;\eta (1,x_{\setminus \{\alpha \}})-\eta (0,x_{\setminus \{\alpha \}})\;\;=\;\eta_{\alpha }(1)-\eta_{\alpha }(0)+\sum_{j\neq \alpha }\{\eta_{\alpha j}(1,x_{j})-\eta_{\alpha j}(0,x_{j})\}\;\;=\;2\eta_{\alpha }(1)+2\sum_{j\neq \alpha }\eta_{\alpha j}(1,x_{j})\;\;≜\;\varsigma^{†}+\sum_{j\neq \alpha }\eta_{j}^{†}(x_{j})$$

where the third equation comes from the SS ANOVA model (29), and fourth equation is the consequence of the side conditions: $\eta_{\alpha }(1)+\eta_{\alpha }(0)=0$ and $\eta_{\alpha j}(1,x_{j})+\eta_{\alpha j}(0,x_{j})=0$ for j≠α. Therefore, $\eta_{\alpha j}(x_{\alpha },x_{j})=0$ (i.e. there is no edge between $x_{\alpha }$ and $x_{j}$) is equivalent to $\eta_{j}^{†}(x_{j})=0$. Consequently, we can consider a logistic regression model with the following model space for the function ν,

(33)
$${ℳ}_{\alpha }^{†}=\{1\}⊕\left\{\underset{j\neq \alpha }{⊕}{ℋ}_{\left(j\right)}^{†}\right\},$$

where $\eta_{j}^{†}\in {ℋ}_{\left(j\right)}^{†}$.

We write the conditional density $f(x_{\alpha }|x_{\setminus \{\alpha \}})=\text{exp}\{x_{\alpha }\nu (x)-\log(1+e^{\nu (x)})\}$. Then, the penalized log-likelihood function

(34)
$$-\frac{1}{n}\sum_{i=1}^{n}\left\{x_{\alpha ,i}\nu (x_{i})-\log(1+e^{\nu (x_{i})})\right\}+\frac{\lambda_{1}}{2}\sum_{j\neq \alpha }\|\eta_{j}^{†}\|.$$

Similar to Zhang et al. (2011), instead of (34), we will minimize the following equivalent but more convenient form

(35)
$$-\frac{1}{n}\sum_{i=1}^{n}\left\{x_{\alpha ,i}\nu (x_{i})-\log(1+e^{\nu (x_{i})})\right\}+\frac{\lambda_{1}}{2}\sum_{j\neq \alpha }\theta_{j}^{-1}{\left\|\eta_{j}^{†}\right\|}^{2}+\lambda_{2}\sum_{j\neq \alpha }\theta_{j}.$$

We approximate the solution as described in Section 3. Denote the approximate solution as $\hat{\nu }(x)=d+\sum_{u=1}^{q}c_{u}\left\{\sum_{j\neq \alpha }\theta_{j}\xi_{ju}(x_{j})\right\}=d+\xi^{T}(x)c$, where d∈R, $\xi_{ju}(x_{j})\;=\;R_{j}({\tilde{x}}_{u,j},x_{j})$ and $\xi (x)={\left(\xi_{1}(x),\cdots ,\xi_{q}(x)\right)}^{T}$. Then, (35) reduces to

(36)
$$-\frac{1}{n}\sum_{i=1}^{n}\left\{x_{\alpha ,i}(d+\xi_{i}^{T}c)-\log(1+e^{d+\xi_{i}^{T}c})\right\}+\frac{\lambda_{1}}{2}c^{T}Qc+\lambda_{2}1^{T}\theta ,$$

subject to θ≥0, where $\xi_{i}=\xi (x_{i})$, $Q={\left\{\sum_{j\neq \alpha }\theta_{j}R_{j}({\tilde{x}}_{u,j},{\tilde{x}}_{v,j})\right\}}_{u,v=1}^{q}$ are defined similarly as in Section 3, 1 is a p−1 vector with all 1’s, and $\theta ={\left(\theta_{1},\cdots ,\theta_{\alpha -1},\theta_{\alpha +1},\cdots ,\theta_{p}\right)}^{T}$.

We need to compute c, d, and θ as minimizers of (36). Again, as the Algorithm in Section 3, we estimate c, d, and θ alternatively. With fixed θ, dropping the last term which is independent of c and d, (36) has the same form as (5.1) in Gu (2013). Therefore, we update c and d using the Newton-Raphson procedure with $\lambda_{1}$ selected by the generalized approximate cross-validation (GACV) method (Gu, 2013).

With fixed c and d, we rewrite $\hat{\nu }(x)=d+\psi^{T}(x)\theta$, where $\psi^{T}(x)=(\psi_{1}^{†},\cdots ,\psi_{\alpha -1}^{†},\psi_{\alpha +1}^{†},\cdots ,\psi_{p}^{†})$ and $\psi_{j}^{†}=\sum_{u=1}^{q}c_{u}\xi_{ju}(x_{j})$. Plugging $\hat{\nu }(x_{i})$ into (35) and keeping terms involving θ only, we have

(37)
$$-\frac{1}{n}\sum_{i=1}^{n}\left\{x_{\alpha ,i}\psi_{i}^{T}\theta -\log(1+e^{d+\psi_{i}^{T}\theta })\right\}+\frac{\lambda_{1}}{2}c^{T}Qc+\lambda_{2}1^{T}\theta ,$$

where $\psi_{i}=\psi (x_{i})$. Furthermore, minimizing (37) is equivalent to minimizing

(38)
$$A(\theta )=-\frac{1}{n}\sum_{i=1}^{n}\left\{x_{\alpha ,i}\psi_{i}^{T}\theta -\log(1+e^{d+\psi_{i}^{T}\theta })\right\}+\frac{\lambda_{1}}{2}c^{T}Qc,$$

subject to θ≥0 and $1^{T}\theta \leq M$ for some constant M. It is easy to see that the Hessian matrix of A(θ) is semi-definite, and consequently, A(θ) is a convex function of θ. We solve (38) iteratively using quadratic programming. Denote the current estimate of θ as $\hat{\theta }$, $\hat{\nu }(x)=d+\psi^{T}(x)\hat{\theta }$, and ${\hat{\nu }}_{i}=\hat{\nu }(x_{i})=d+\psi_{i}^{T}\hat{\theta }$. We update θ by minimizing the following second-order Taylor approximation of A(θ) (some constants independent of θ have been removed):

(39)
$$\frac{1}{2}\theta^{T}H_{A}(\tilde{\theta })\theta +\theta^{T}\left\{G_{A}(\tilde{\theta })-H_{A}(\tilde{\theta })\tilde{\theta }\right\}$$

subject to θ≥0 and $1^{T}\theta \leq M$ for some constant M, where $G_{A}(\tilde{\theta })=-n^{-1}\sum_{i=1}^{n}\{x_{\alpha ,i}\psi_{i}-\psi_{i}e^{{\hat{\nu }}_{i}}∕(1+e^{{\hat{\nu }}_{i}})\}+\lambda_{1}q∕2$ is the gradient, $H_{A}(\tilde{\theta })=\frac{1}{n}\sum_{i=1}^{n}\psi_{i}\psi_{i}^{T}{e^{{\hat{\nu }}_{i}}∕(1+e^{{\hat{\nu }}_{i}})}^{2}$ is the Hessian, $q={\left(c^{T}Q_{1}c,\cdots ,c^{T}Q_{\alpha -1}c,\cdots ,c^{T}Q_{\alpha +1}c,\cdots ,c^{T}Q_{p}c\right)}^{T}$, and $Q_{j}={\left\{R_{j}({\tilde{x}}_{u,j},{\tilde{x}}_{v,j})\right\}}_{u,v=1}^{q}$ for j=1,⋯,p and j≠α. We select the tuning parameter M by the K-fold cross-validation or the BIC method. We skip the implementation detail for the above algorithm since it is similar to that for the pseudo log-likelihood approach described in the next section.

## Appendix B. Algorithm Implementation

In this section, we provide details about the implementation of the proposed algorithm using existing R packages. Specifically, we implement the Newton-Raphson procedure in the algorithm using a modification of the sscden1 function in the gss package (Gu et al., 2014) and quadratic programming using the R function solve.QP in the quadprog package (Turlach and Weingessel, 2007).

### B.1 Implementation of the Newton-Raphson Method

Given the current value of $\theta_{2}$, we update c and d by minimizing (13) using the Newton-Raphson method. We implement by modifying the function sscden1 in the gss package since (13) has the same form as (10.31) in Gu (2013) with different penalties and certain smoothing parameters being fixed. By definition, ${ℋ}_{\left(\alpha k\right)}={ℋ}_{\left(\alpha \right)}⊗{ℋ}_{\left(k\right)}=({ℋ}_{\left(\alpha \right)}^{0}⊕{ℋ}_{\left(\alpha \right)}^{1})⊗({ℋ}_{\left(k\right)}^{0}⊕{ℋ}_{\left(k\right)}^{1})=({ℋ}_{\left(\alpha \right)}^{0}⊗{ℋ}_{\left(k\right)}^{0})⊕({ℋ}_{\left(\alpha \right)}^{0}⊗{ℋ}_{\left(k\right)}^{1})⊕({ℋ}_{\left(\alpha \right)}^{1}⊗{ℋ}_{\left(k\right)}^{0})⊕({ℋ}_{\left(\alpha \right)}^{1}⊗{ℋ}_{\left(k\right)}^{1})={ℋ}_{\left(\alpha k\right)}^{\left(0\right)}⊕{ℋ}_{\left(\alpha k\right)}^{\left(1\right)}⊕{ℋ}_{\left(\alpha k\right)}^{\left(2\right)}⊕{ℋ}_{\left(\alpha k\right)}^{\left(3\right)}$ where ${ℋ}_{\left(\alpha k\right)}^{\left(0\right)}={ℋ}_{\left(\alpha \right)}^{0}⊗{ℋ}_{\left(k\right)}^{0}$, ${ℋ}_{\left(\alpha k\right)}^{\left(1\right)}={ℋ}_{\left(\alpha \right)}^{0}⊗{ℋ}_{\left(k\right)}^{1}$, ${ℋ}_{\left(\alpha k\right)}^{\left(2\right)}={ℋ}_{\left(\alpha \right)}^{1}⊗{ℋ}_{\left(k\right)}^{0}$, and ${ℋ}_{\left(\alpha k\right)}^{\left(3\right)}={ℋ}_{\left(\alpha \right)}^{1}⊗{ℋ}_{\left(k\right)}^{1}$. For density estimation, the penalized log-likelihood method in Gu (2013) does not penalize functions in the parametric component space ${ℋ}_{\left(\alpha k\right)}^{0}$ and has different smoothing parameters for components in the nonparametric component spaces ${ℋ}_{\left(\alpha k\right)}^{\left(1\right)}$, ${ℋ}_{\left(\alpha k\right)}^{\left(2\right)}$, and ${ℋ}_{\left(\alpha k\right)}^{\left(3\right)}$. Our goal is edge detection by detecting nonzero interactions. Therefore, we penalize the combined interaction $\eta_{\alpha k}\in {ℋ}_{\left(\alpha k\right)}$ as a whole with a smoothing parameter $\theta_{\alpha k}$ for k=1,⋯,p and k≠α. The interaction $\eta_{\alpha k}$ collects parametric and nonparametric interaction components in ${ℋ}_{\left(\alpha k\right)}^{\left(0\right)}$, ${ℋ}_{\left(\alpha k\right)}^{\left(1\right)}$, ${ℋ}_{\left(\alpha k\right)}^{\left(2\right)}$, and ${ℋ}_{\left(\alpha k\right)}^{\left(3\right)}$. Note that $\theta_{2}={\left(\theta_{\alpha 1},\cdots ,\theta_{\alpha (\alpha -1)},\theta_{\alpha (\alpha +1)},\cdots ,\theta_{\alpha p}\right)}^{T}$ is fixed at this step. We modified the function sscden1 to solve (13) with smoothing parameters $\lambda_{1}$ and $\theta_{1}$ estimated by the approximated cross-validation method.

### B.2 Implementation of Quadratic Programming

Denote the current estimate of $\theta_{2}$ as ${\tilde{\theta }}_{2}$ and $\tilde{g}(x)=\phi^{T}(x)d+\psi_{1}^{T}(x)\theta_{1}+\psi_{2}^{T}(x){\tilde{\theta }}_{2}$. Define $\mu_{\tilde{g}}(h)=\sum_{i=1}^{n}e^{-\tilde{g}(x_{i})}h(x_{i})∕\sum_{i=1}^{n}e^{-\tilde{g}(x_{i})}$, $V_{\tilde{g}}(h_{1},h_{2})=\mu_{\tilde{g}}(h_{1},h_{2})-\mu_{\tilde{g}}(h_{1})\mu_{\tilde{g}}(h_{2})$ for any functions h, $h_{1}$, and $h_{2}$. We update $\theta_{2}$ by minimizing the following second-order Taylor approximation of $A_{2}(\theta_{2})$ with some constants independent of $\theta_{2}$ have been removed:

(40)
$$\frac{1}{2}\theta_{2}^{T}H_{A}({\tilde{\theta }}_{2})\theta_{2}+\theta_{2}^{T}\left\{G_{A}({\tilde{\theta }}_{2})-H_{A}({\tilde{\theta }}_{2}){\tilde{\theta }}_{2}\right\}$$

subject to $\theta_{2}\geq 0$ and $w^{T}\theta_{2}\leq M$ for some constant M, where $G_{A}({\tilde{\theta }}_{2})=-\mu_{\tilde{g}}(\psi_{2})+b_{\psi_{2}}+\lambda_{1}q_{2}∕2$ is the gradient, $H_{A}({\tilde{\theta }}_{2})=V_{\tilde{g}}(\psi_{2},\psi_{2}^{T})$ is the Hessian, $q_{2}={\left(w_{\alpha 1}^{-1}c^{T}Q_{\alpha 1}c,\cdots ,w_{\alpha (\alpha -1)}^{-1}c^{T}Q_{\alpha (\alpha -1)}c,w_{\alpha (\alpha +1)}^{-1}c^{T}Q_{\alpha (\alpha +1)}c,\cdots ,w_{\alpha p}^{-1}c^{T}Q_{\alpha p}c\right)}^{T}$, and $Q_{\alpha k}={\left\{R_{\alpha k}(({\tilde{x}}_{u,\alpha },{\tilde{x}}_{u,k})({\tilde{x}}_{v,\alpha },{\tilde{x}}_{v,k}))\right\}}_{u,v=1}^{q}$ for k=1,⋯,p and k≠α.

We use the R function solve.QP to solve (40). We estimate the tuning parameter M by minimizing a K-fold cross-validation or BIC score defined as follows. Let $I_{1},\cdots ,I_{K}$ be K randomly partitioned subsamples of the original data, $n_{j}=|I_{j}|$, and $n_{\left(-j\right)}=n-n_{j}$. Denote $g_{M}^{\left(-j\right)}$ as the estimate without observations in the subset $I_{j}$ which minimizes the following function with respect to $\theta_{2}$:

(41)
$$\log\left\{\frac{1}{n_{\left(-j\right)}}\sum_{i\notin I_{j}}e^{-g(x_{i}^{\alpha })}\right\}+\frac{1}{n_{\left(-j\right)}}\sum_{i\notin I_{j}}\int_{{𝒳}_{\alpha }}g(x_{i}^{\alpha })\rho (x_{i}^{\alpha })dx_{\alpha }+\lambda_{1}\sum_{k\neq \alpha }w_{\alpha k}\theta_{\alpha k}^{-1}{\left\|\eta_{\alpha k}\right\|}^{2}$$

subject to $\theta_{\alpha k}\geq 0$ for k≠α and $w^{T}\theta_{2}\leq M$. The K-fold cross-validation estimate of M is the minimizer of the following score:

(42)
$$\text{CV}(M)=\log\left\{\frac{1}{n}\sum_{j=1}^{K}\sum_{i\in I_{j}}e^{-g_{M}^{\left(-j\right)}(x_{i}^{\alpha })}\right\}+\frac{1}{n}\sum_{j=1}^{K}\sum_{i\in I_{j}}\int_{{𝒳}_{\alpha }}g_{M}^{\left(-j\right)}(x_{i}^{\alpha })\rho (x_{i}^{\alpha })dx_{\alpha }.$$

The BIC estimate of M is the minimizer of the following score:

(43)
$$\text{BIC}(M)=\log\left\{\frac{1}{n}\sum_{i=1}^{n}e^{-g_{M}(x_{i}^{\alpha })}\right\}+\frac{1}{n}\sum_{i=1}^{n}\int_{{𝒳}_{\alpha }}g_{M}(x_{i}^{\alpha })\rho (x_{i}^{\alpha })dx_{\alpha }+\log(nk_{n}),$$

where $g_{M}$ expresses the dependence of the estimate on M explicitly, and $k_{n}$ is the number of nonzero elements in the estimate of $\theta_{2}$. We applied the K-fold cross-validation method in all simulations with K=5. We applied the BIC method in real data examples to get sparser graphs.

### B.3 Initial Values and Convergence Criterion

To get a good initial value $\theta_{2,0}$, we first estimate the conditional density $f(x_{\alpha }|x_{\setminus \{\alpha \}})$ with $\tau_{1}\sum_{k\neq \alpha }w_{\alpha k}\|\eta_{\alpha k}\|$ in (7) being replaced by $(\lambda_{1}∕2)\sum_{k\neq \alpha }\theta_{\alpha k}^{-1}{\left\|\eta_{\alpha k}\right\|}^{2}$. We modified the sscden function in the gss package to estimate the conditional density and denote the estimate of $\eta_{\alpha k}$ as ${\overset{ˇ}{\eta }}_{\alpha k}$. Since $\theta_{\alpha k}\;=\;0$ in $\theta_{2}$ iff $\eta_{\alpha k}\;=\;0$, the magnitude of ${\overset{ˇ}{\eta }}_{\alpha k}$ provides one way to initialize $\theta_{\alpha k}$. Specifically, we set $\theta_{\alpha k}^{0}={\left\{\sum_{i=1}^{n}{\overset{ˇ}{\eta }}_{\alpha k}^{2}(x_{i})\right\}}^{1∕2}$.

The convergence criterion in the algorithm is ${\left\|\theta_{2}-{\tilde{\theta }}_{2}\right\|}_{2}∕({\left\|{\tilde{\theta }}_{2}\right\|}_{2}+10^{-6})\leq \varepsilon$ or the number of zeros in $\theta_{2}$ stops increasing for fixed number of steps, where $\theta_{2}$ and ${\tilde{\theta }}_{2}$ are the updated and previous estimates, respectively, ${\left\|\cdot \right\|}_{2}$ is the Euclidean norm, and ε a threshold. We set ε=0.001 in simulation and real data examples.

## Appendix C. Proofs

**Proof of** Proposition 1: We show the equivalence between minimization problems (7) and (9). First,

(44)
minη∈ℳα{1n∑i=1n{e−η(xi)+∫𝒳αη(xiα)ρ(xiα)dxα}+λ12∑j=1pθj−1‖Pjηj‖2+τ1∑k≠αwαk‖ηαk‖}=ming∈𝒢,ς∈R{1n∑i=1n{e−g(xi)−ς+∫𝒳α(g(xiα)+ς)ρ(xiα)dxα}+λ12∑j=1pθj−1‖Pjηj‖2}{+τ1∑k≠αwαk‖ηαk‖}.

Setting the derivative of (44) with respect to ς to zero, we get $e^{\varsigma }=n^{-1}\sum_{i=1}^{n}e^{-g(x_{i})}$. Plugging it back to (44), we have the following profiled penalized pseudo log-likelihood

ming∈𝒢{1+1n∑i=1n∫𝒳αg(xiα)ρ(xiα)dxα+log{1n∑i=1ne−g(xi)}+λ12∑j=1pθj−1‖Pjηj‖2}{+τ1∑k≠αwαk‖ηαk‖}.

□

**Proof of** Proposition 2: Set $\lambda_{2}=\tau_{1}^{2}∕2\lambda_{1}$. Denote the functional in (9) as $B_{1}(g)$ and the functional in (10) as $B_{2}(\theta_{2},g)$. For any $\theta_{\alpha k}\geq 0$ and g∈𝒢, we have $\lambda_{1}\theta_{\alpha k}^{-1}{\left\|\eta_{\alpha k}\right\|}^{2}∕2+\lambda_{2}\theta_{\alpha k}\geq \sqrt{2}\lambda_{1}^{1∕2}\lambda_{2}^{1∕2}\|\eta_{\alpha k}\|=\tau_{1}\|\eta_{\alpha k}\|$, and the equality holds if and only if $\theta_{\alpha k}=\lambda_{1}^{1∕2}\lambda_{2}^{-1∕2}\|\eta_{\alpha k}\|∕\sqrt{2}$. Therefore, $B_{2}(\theta_{2},g)\geq B_{1}(g)$ for any $\theta_{\alpha k}\geq 0$ and g∈𝒢, and the equality holds if and only if $\theta_{\alpha k}=\lambda_{1}^{1∕2}\lambda_{2}^{-1∕2}\|\eta_{\alpha k}\|∕\sqrt{2}$ for α≠k. The equivalence between (9) and (10) follows. □

**Proof of** Proposition 3: The population version of the pseudo log-likelihood $l_{\alpha }=n^{-1}\sum_{i=1}^{n}\left\{e^{-\eta (x_{i})}+\int_{{𝒳}_{\alpha }}\eta (x_{i}^{\alpha })\rho (x_{i}^{\alpha })dx_{\alpha }\right\}$ in (7) is

(45)
$$l(\eta )=E[e^{-\eta (x)}]+\int_{{𝒳}_{\setminus \{\alpha \}}}f_{\setminus \{\alpha \}}(x_{\setminus \{\alpha \}})\int_{{𝒳}_{\alpha }}\eta (x)\rho (x)dx\;\;=\int_{{𝒳}_{\setminus \{\alpha \}}}f_{\setminus \{\alpha \}}(x_{\setminus \{\alpha \}})\int_{{𝒳}_{\alpha }}e^{-\eta (x)}f(x_{\alpha }|x_{\setminus \{\alpha \}})dx+\int_{{𝒳}_{\setminus \{\alpha \}}}f_{\setminus \{\alpha \}}(x_{\setminus \{\alpha \}})\int_{{𝒳}_{\alpha }}\eta (x)\rho (x)dx,$$

where $f_{\setminus \{\alpha \}}(x_{\setminus \{\alpha \}})$ is the density of $X_{\setminus \{\alpha \}}$ on ${𝒳}_{\setminus \{\alpha \}}={𝒳}_{1}\times \cdots \times {𝒳}_{\alpha -1}\times {𝒳}_{\alpha +1}\times \cdots \times {𝒳}_{p}$. The first and second-order Fréchet derivatives of l(η) are

$$\;Dl(\eta )h_{1}=-\int_{{𝒳}_{\setminus \{\alpha \}}}f_{\setminus \{\alpha \}}(x_{\setminus \{\alpha \}})\int_{{𝒳}_{\alpha }}e^{-\eta (x)}h_{1}(x)f(x_{\alpha }|x_{\setminus \{\alpha \}})dx\;\;+\int_{{𝒳}_{\setminus \{\alpha \}}}f_{\setminus \{\alpha \}}(x_{\setminus \{\alpha \}})\int_{{𝒳}_{\alpha }}h_{1}(x)\rho (x)dx\;D^{2}l(f)h_{1}h_{2}=\int_{{𝒳}_{\setminus \{\alpha \}}}f_{\setminus \{\alpha \}}(x_{\setminus \{\alpha \}})\int_{{𝒳}_{\alpha }}e^{-\eta (x)}h_{1}(x)h_{2}(x)f(x_{\alpha }|x_{\setminus \{\alpha \}})dx,$$

where D denotes Frédiet derivative operator.

We set $Dl(\eta )h_{1}=\int_{{𝒳}_{\setminus \{\alpha \}}}f_{\setminus \{\alpha \}}(x_{\setminus \{\alpha \}})\int_{{𝒳}_{\alpha }}h_{1}(x)[\rho (x)-e^{-\eta (x)}f(x_{\alpha }|x_{\setminus \{\alpha \}})]dx=0$ for all $h_{1}\in {ℳ}_{\alpha }$. Then, we have $\rho (x)-e^{-\eta (x)}f(x_{\alpha }|x_{\setminus \{\alpha \}})=0$, which implies that $f(x_{\alpha }|x_{\setminus \{\alpha \}})=e^{\eta (x)}\rho (x)$. In addition, $D^{2}l(\eta )hh=\int_{{𝒳}_{\setminus \{\alpha \}}}f_{\setminus \{\alpha \}}(x_{\setminus \{\alpha \}})\int_{{𝒳}_{\alpha }}e^{-\eta (x)}h^{2}(x)f(x_{\alpha }|x_{\setminus \{\alpha \}})dx>0$ for any nonzero $h\in {ℳ}_{\alpha }$, and consequently, l is strictly convex. Therefore, if $\hat{\eta }$ is the solution to (7), the estimate of the conditional density equals $e^{\hat{\eta }(x)}\rho (x)$.

We note that $\hat{\eta }=\hat{\varsigma }+\hat{g}$ where $\hat{\eta }$ is the solution to (7), $\hat{\varsigma }$ is given in Proposition 1, and $\hat{g}$ is the solution to (10). Since $\hat{\varsigma }$ is a constant independent of $x_{\alpha }$, then the estimate of the conditional density $\hat{f}(x_{\alpha }|x_{\setminus \{\alpha \}})$ is proportional to $e^{\hat{g}(x)}\rho (x)$. □

**Proof of Convexity of** $A_{2}(\theta_{2})$: We show that the Hessian matrix $H_{A}(\theta_{2})$ of $A_{2}(\theta_{2})$ is positive semi-definite. For any vector ν≠0, let $s_{i}=e^{-g(x_{i})}$ and $t_{i}=\nu^{T}\psi_{2}(x_{i})$, we have

(46)
$$\nu^{T}H_{A}(\theta_{2})\nu =\frac{\left(\sum_{i=1}^{n}s_{i}t_{i}^{2}\right)\left(\sum_{i=1}^{n}s_{i}\right)-{\left(\sum_{i=1}^{n}t_{i}s_{i}\right)}^{2}}{{\left(\sum_{i=1}^{n}s_{i}\right)}^{2}}\geq 0,$$

by the Cauchy-Schwartz inequality. □

In the remainder of the Appendix, we first introduce three lemmas and then provide proofs of Theorem 1, Theorem 2, and Corollary 1.

**Lemma 1** *Assume* $J^{*}(g_{0})<\infty$. *Under* Conditions 1-3*, as* $\lambda_{1}\to 0$ *and* n→∞,

$$(V^{*}+\lambda_{1}J^{*})(\tilde{g}-g_{0})=O_{p}(n^{-1}\lambda_{1}^{-1∕r}+\lambda_{1}).$$

**Proof**: By the Fourier series expansions of $\tilde{g}$ and $g_{0}$, we have

$$\;V^{*}(\tilde{g}-g_{0})=\sum_{v}{\left({\tilde{a}}_{v}-a_{v,0}\right)}^{2}=\sum_{v}\frac{\kappa_{v}^{2}-2\kappa_{v}\lambda_{1}\gamma_{v}a_{v,0}+\lambda_{1}^{2}\gamma_{v}^{2}a_{v,0}^{2}}{{\left(1+\lambda_{1}\gamma_{v}\right)}^{2}},\;\lambda_{1}J^{*}(\tilde{g}-g_{0})=\sum_{v}\lambda_{1}\gamma_{v}{\left({\tilde{a}}_{v}-a_{v,0}\right)}^{2}=\sum_{v}\lambda_{1}\gamma_{v}\frac{\kappa_{v}^{2}-2\kappa_{v}\lambda_{1}\gamma_{v}a_{v,0}+\lambda_{1}^{2}\gamma_{v}^{2}a_{v,0}^{2}}{{\left(1+\lambda_{1}\gamma_{v}\right)}^{2}}.$$

Since $E(\kappa_{v})=0$ and $E(\kappa_{v}^{2})\leq c_{1}∕n$, we have

(47)
$$\;E[V^{*}(\tilde{g}-g_{0})]\leq \frac{c_{1}}{n}\sum_{v}\frac{1}{{\left(1+\lambda_{1}\gamma_{v}\right)}^{2}}+\lambda_{1}\sum_{v}\frac{\lambda_{1}\gamma_{v}}{{\left(1+\lambda_{1}\gamma_{v}\right)}^{2}}\gamma_{v}a_{v,0}^{2},\;E[\lambda_{1}J^{*}(\tilde{g}-g_{0})]\leq \frac{c_{1}}{n}\sum_{v}\frac{\lambda_{1}\gamma_{v}}{{\left(1+\lambda_{1}\gamma_{v}\right)}^{2}}+\lambda_{1}\sum_{v}\frac{{\left(\lambda_{1}\gamma_{v}\right)}^{2}}{{\left(1+\lambda_{1}\gamma_{v}\right)}^{2}}\gamma_{v}a_{v,0}^{2}.$$

Following similar arguments in the proof of Lemma 9.1 in Gu (2013), we have

$$\sum_{v}\frac{\lambda_{1}\gamma_{v}}{{\left(1+\lambda_{1}\gamma_{v}\right)}^{2}}=O(\lambda_{1}^{-1∕r}),\;\;\sum_{v}\frac{1}{{\left(1+\lambda_{1}\gamma_{v}\right)}^{2}}=O(\lambda_{1}^{-1∕r}),\;\;\sum_{v}\frac{1}{1+\lambda_{1}\gamma_{v}}=O(\lambda_{1}^{-1∕r}).$$

The lemma follows from (47) and the fact that $\sum_{v}\gamma_{v}a_{v,0}^{2}=J^{*}(g_{0})<\infty$. □

As in Gu (2013), when $g_{0}$ is “supersmooth” in the sense that $\sum_{v}\gamma_{v}^{l}a_{v,0}^{2}<\infty$ for some 1<l≤2, which is assumed in Theorem 1, the rates can be improved to $O(n^{-1}\lambda_{1}^{-1∕r}+\lambda_{1}^{l}$.

Now we want to bound the approximation error $\hat{g}-\tilde{g}$. Define

$$A_{h_{1},h_{2}}(\tau )=\frac{1}{n}\sum_{i=1}^{n}e^{-(h_{1}+\tau h_{2})(X_{i})}+\frac{1}{n}\sum_{i=1}^{n}\int_{{𝒳}_{\alpha }}(h_{1}+\tau h_{2})\rho (x_{i}^{\alpha })dx_{\alpha }+\frac{\lambda_{1}}{2}J^{*}(h_{1}+\tau h_{2})\;\;+\lambda_{2}\sum_{k\neq \alpha }\theta_{\alpha k},\;B_{h_{1},h_{2}}(\tau )=\frac{1}{n}\sum_{i=1}^{n}-e^{-g_{0}(X_{i})}(h_{1}+\tau h_{2})(X_{i})+\frac{1}{n}\sum_{i=1}^{n}\int_{{𝒳}_{\alpha }}(h_{1}+\tau h_{2})\rho (x_{i}^{\alpha })dx_{\alpha }\;\;+\frac{1}{2}V^{*}(h_{1}+\tau h_{2}-g_{0})+\frac{\lambda_{1}}{2}J^{*}(h_{1}+\tau h_{2}).$$

Taking derivatives of $A_{h_{1},h_{2}}$ and $B_{h_{1},h_{2}}$ with respect to τ and evaluating them at τ=0, we obtain

(48)
$${\dot{A}}_{h_{1},h_{2}}(0)=-\frac{1}{n}\sum_{i=1}^{n}e^{-h_{1}(X_{i})}h_{2}(X_{i})+\frac{1}{n}\sum_{i=1}^{n}\int_{{𝒳}_{\alpha }}h_{2}\rho (x_{i}^{\alpha })dx_{\alpha }+\lambda_{1}J^{*}(h_{1},h_{2}),$$

(49)
$${\dot{B}}_{h_{1},h_{2}}(0)=-\frac{1}{n}\sum_{i=1}^{n}e^{-g_{0}(X_{i})}h_{2}(X_{i})+\frac{1}{n}\sum_{i=1}^{n}\int_{{𝒳}_{\alpha }}h_{2}\rho (x_{i}^{\alpha })dx_{\alpha }+V^{*}(h_{1}-g_{0},h_{2})+\lambda_{1}J^{*}(h_{1},h_{2}).$$

Setting $h_{1}=\hat{g}$ and $h_{2}=\hat{g}-\tilde{g}$ in (48), we have

(50)
$$-\frac{1}{n}\sum_{i=1}^{n}e^{-\hat{g}(X_{i})}(\hat{g}-\tilde{g})(X_{i})+\frac{1}{n}\sum_{i=1}^{n}\int_{{𝒳}_{\alpha }}(\hat{g}-\tilde{g})\rho (x_{i}^{\alpha })dx_{\alpha }+\lambda_{1}J^{*}(\hat{g},\hat{g}-\tilde{g})=0.$$

Setting $h_{1}=\tilde{g}$ and $h_{2}=\hat{g}-\tilde{g}$ in (49), we have

(51)
$$-\frac{1}{n}\sum_{i=1}^{n}e^{-g_{0}(X_{i})}(\hat{g}-\tilde{g})(X_{i})+\frac{1}{n}\sum_{i=1}^{n}\int_{{𝒳}_{\alpha }}(\hat{g}-\tilde{g})\rho (x_{i}^{\alpha })dx_{\alpha }+V^{*}(\tilde{g}-g_{0},\hat{g}-\tilde{g})\;+\lambda_{1}J^{*}(\tilde{g},\hat{g}-\tilde{g})=0.$$

Subtracting (51) from (50), we obtain

(52)
$$\;\lambda_{1}J^{*}(\hat{g}-\tilde{g})-\frac{1}{n}\sum_{i=1}^{n}\left\{e^{-\hat{g}(X_{i})}-e^{-\tilde{g}(X_{i})}\right\}(\hat{g}-\tilde{g})(X_{i})\;=\frac{1}{n}\sum_{i=1}^{n}\left\{e^{-\tilde{g}(X_{i})}-e^{-g_{0}(X_{i})}\right\}(\hat{g}-\tilde{g})(X_{i})+V^{*}(\hat{g}-\tilde{g},\tilde{g}-g_{0}).$$

Applying the mean value theorem, we have $e^{-\hat{g}(X_{i})}-|e^{-\tilde{g}(X_{i})}\;=\;-e^{-(\tilde{g}+\tau_{i}(\hat{g}-\tilde{g}))(X_{i})}(\hat{g}-\tilde{g})(X_{i})$ where $\tau_{i}\in [0,1]$. Since $\hat{g}$ and $\tilde{g}$ belong to $B_{0}$ which is a convex set around $g_{0}$, under Condition 4, there exists a $b_{0}^{\left(i\right)}\in (c_{2},c_{3})$ such that $-e^{-(\tilde{g}+\tau_{i}(\hat{g}-\tilde{g}))(X_{i})}(\hat{g}-\tilde{g})(X_{i})=-b_{0}^{\left(i\right)}e^{-g_{0}(X_{i})}(\hat{g}-\tilde{g})(X_{i})$. Then

(53)
$$-\frac{1}{n}\sum_{i=1}^{n}\left\{e^{-\hat{g}(X_{i})}-e^{-\tilde{g}(X_{i})}\right\}(\hat{g}-\tilde{g})(X_{i})=\frac{1}{n}\sum_{i=1}^{n}b_{0}^{\left(i\right)}e^{-g_{0}(X_{i})}{\left(\hat{g}-\tilde{g}\right)}^{2}(X_{i})\;\;\geq \frac{c_{2}}{n}\sum_{i=1}^{n}e^{-g_{0}(X_{i})}{\left(\hat{g}-\tilde{g}\right)}^{2}(X_{i}).$$

By the same argument, there exists a $c_{0}^{\left(i\right)}\in (c_{2},c_{3})$ such that

(54)
$$\frac{1}{n}\sum_{i=1}^{n}\left\{e^{-\tilde{g}(X_{i})}-e^{-g_{0}(X_{i})}\right\}(\hat{g}-\tilde{g})(X_{i})=-\frac{1}{n}\sum_{i=1}^{n}c_{0}^{\left(i\right)}e^{-g_{0}(X_{i})}(\hat{g}-\tilde{g})(X_{i})(\tilde{g}-g_{0})(X_{i}).$$

**Lemma 2** *Under Conditions 1, 2, and 5, suppose* $h_{1}$ *and* $h_{2}$ *are functions satisfying* $J^{*}(h_{1})<\infty$ *and* $J^{*}(h_{2})<\infty$*, as* $\lambda_{1}\to 0$ *and* $n\lambda_{1}^{2∕r}\to \infty$*, one has*

(55)
$$|\frac{1}{n}\sum_{i=1}^{n}e^{-g_{0}(X_{i})}h_{1}(X_{i})h_{2}(X_{i})-V^{*}(h_{1},h_{2})|=o_{p}\left({\left\{(V^{*}+\lambda_{1}J^{*})(h_{1})(V^{*}+\lambda_{1}J^{*})(h_{2})\right\}}^{1∕2}\right).$$

**Proof**: Since $J^{*}(h_{1})<\infty$ and $J^{*}(h_{2})<\infty$, then $h_{1}$ and $h_{2}$ can be expressed as Fourier series $h_{1}=\sum_{v}h_{1,v}\zeta_{v}$ and $h_{2}=\sum_{v}h_{2,v}\zeta_{v}$. Let

$$U_{i}=\zeta_{v}(X_{i})\zeta_{u}(X_{i})e^{-g_{0}(X_{i})}-\int_{{𝒳}_{\setminus \{\alpha \}}}f_{\setminus \{\alpha \}}(x_{\setminus \{\alpha \}})\int_{{𝒳}_{\alpha }}\zeta_{v}(x)\zeta_{u}(x)\rho (x)dx_{\alpha }dx_{\setminus \{\alpha \}}.$$

Note that $U_{i}$ are i.i.d. random variables with $E(U_{i})=0$. Then under Condition 5, we have

$$E{\left(\frac{1}{n}\sum_{i=1}^{n}U_{i}\right)}^{2}=\frac{1}{n}\text{Var}\left(\zeta_{v}(X_{1})\zeta_{u}(X_{1})e^{-g_{0}(X_{1})}\right)<\frac{c_{4}}{n}.$$

Furthermore,

$$\;|\frac{1}{n}\sum_{i=1}^{n}e^{-g_{0}(X_{i})}h_{1}(X_{i})h_{2}(X_{i})-V^{*}(h_{1},h_{2})|\;=|\sum_{v}\sum_{u}h_{1,v}h_{2,u}\frac{1}{n}\sum_{i=1}^{n}U_{i}|\;\leq {\left\{\sum_{v}\sum_{u}\frac{1}{1+\lambda_{1}\gamma_{v}}\frac{1}{1+\lambda_{1}\gamma_{u}}{\left(\frac{1}{n}\sum_{i=1}^{n}U_{i}\right)}^{2}\right\}}^{1∕2}{\left\{\sum_{v}\sum_{u}(1+\lambda_{1}\gamma_{v})(1+\lambda_{1}\gamma_{u})h_{1,v}^{2}h_{2,u}^{2}\right\}}^{1∕2}\;=O_{p}(n^{-1∕2}\lambda_{1}^{-1∕r}){\left\{(V^{*}+\lambda_{1}J^{*})(h_{1})(V^{*}+\lambda_{1}J^{*})(h_{2})\right\}}^{1∕2}\;=o_{p}\left({\left\{(V^{*}+\lambda_{1}J^{*})(h_{1})(V^{*}+\lambda_{1}J^{*})(h_{2})\right\}}^{1∕2}\right),$$

where the second equality holds because of the fact $\sum_{v}\frac{1}{1+\lambda_{1}\gamma_{v}}=O(\lambda_{1}^{-1∕r})$ and the strong law of large numbers. □

**Lemma 3** *Under* Conditions 1, 2, and 5*, as* $\lambda_{1}\to 0$ *and* $n\lambda_{1}^{2∕r}\to \infty$*, then*

(56)
$$|\frac{1}{n}\sum_{i=1}^{n}e^{-g_{0}(X_{i})}h_{1}(X_{i})h_{2}(X_{i})-\frac{1}{n}\sum_{i=1}^{n}c_{0}^{\left(i\right)}e^{-g_{0}(X_{i})}h_{1}(X_{i})h_{2}(X_{i})|\;\;\leq 2c_{0}{\left\{(V^{*}+\lambda_{1}J^{*})(h_{1})(V^{*}+\lambda_{1}J^{*})(h_{2})\right\}}^{1∕2}$$

*holds with probability 1, where* $c_{0}=max\{|c_{2}-1|,|c_{3}-1|\}$.

**Proof**: Note that

$$\;E|e^{-g_{0}(X_{i})}h_{1}(X_{i})h_{2}(X_{i})|\;=\int_{{𝒳}_{\setminus \{\alpha \}}}f_{\setminus \{\alpha \}}(x_{\setminus \{\alpha \}})\int_{{𝒳}_{\alpha }}|h_{1}(x)h_{2}(x)|\rho (x)dx_{\alpha }dx_{\setminus \{\alpha \}}\;\leq {\left\{\left(\int_{{𝒳}_{\setminus \{\alpha \}}}f_{\setminus \{\alpha \}}(x_{\setminus \{\alpha \}})\int_{{𝒳}_{\alpha }}h_{1}^{2}(x)\rho (x)dx_{\alpha }dx_{\setminus \{\alpha \}})(\int_{{𝒳}_{\setminus \{\alpha \}}}f_{\setminus \{\alpha \}}(x_{\setminus \{\alpha \}})\int_{{𝒳}_{\alpha }}h_{2}^{2}(x)\rho (x)dx_{\alpha }dx_{\setminus \{\alpha \}}\right)\right\}}^{1∕2}\;={\left\{V^{*}(h_{1})V^{*}(h_{2})\right\}}^{1∕2}\;\leq {\left\{(V^{*}+\lambda_{1}J^{*})(h_{1})(V^{*}+\lambda_{1}J^{*})(h_{2})\right\}}^{1∕2},$$

where the first inequality follows the Cauchy-Schwartz inequality. Then, we have

$$\;|\frac{1}{n}\sum_{i=1}^{n}e^{-g_{0}(X_{i})}h_{1}(X_{i})h_{2}(X_{i})-\frac{1}{n}\sum_{i=1}^{n}c_{0}^{\left(i\right)}e^{-g_{0}(X_{i})}h_{1}(X_{i})h_{2}(X_{i})|\;=|\frac{1}{n}\sum_{i=1}^{n}(1-c_{0}^{\left(i\right)})e^{-g_{0}(X_{i})}h_{1}(X_{i})h_{2}(X_{i})|\;\leq \frac{1}{n}\sum_{i=1}^{n}|(1-c_{0}^{\left(i\right)})∥e^{-g_{0}(X_{i})}h_{1}(X_{i})h_{2}(X_{i})|\;\leq c_{0}\frac{1}{n}\sum_{i=1}^{n}|e^{-g_{0}(X_{i})}h_{1}(X_{i})h_{2}(X_{i})|\;\leq 2c_{0}{\left\{(V^{*}+\lambda_{1}J^{*})(h_{1})(V^{*}+\lambda_{1}J^{*})(h_{2})\right\}}^{1∕2},$$

where the last inequality holds due to the strong law of large numbers. □

**Proof of** Theorem 1: Note that $E\{e^{-g_{0}(X_{i})}{\left(\hat{g}-\tilde{g}\right)}^{2}(X_{i})\}=\int_{{𝒳}_{\setminus \{\alpha \}}}f_{\setminus \{\alpha \}}(x_{\setminus \{\alpha \}})\int_{{𝒳}_{\alpha }}{\left(\hat{g}-\tilde{g}\right)}^{2}(x)\rho (x)dx_{\alpha }dx_{\setminus \{\alpha \}}=V^{*}(\hat{g}-\tilde{g})$. Substituting (53) into the left-hand side of (52), we have

(57)
$$\;\lambda_{1}J^{*}(\hat{g}-\tilde{g})-\frac{1}{n}\sum_{i=1}^{n}\left\{e^{-\hat{g}(X_{i})}-e^{-\tilde{g}(X_{i})}\right\}(\hat{g}-\tilde{g})(X_{i})\;\geq \frac{c_{2}}{n}\sum_{i=1}^{n}e^{-g_{0}(X_{i})}{\left(\hat{g}-\tilde{g}\right)}^{2}(X_{i})+\lambda_{1}J^{*}(\hat{g}-\tilde{g})\;\geq \frac{c_{2}}{2}V^{*}(\hat{g}-\tilde{g})+\lambda_{1}J^{*}(\hat{g}-\tilde{g}),$$

where the last equality holds due to the strong law of large numbers. Substituting (55) and (56) into the right-hand side of (52) and letting $h_{1}=\hat{g}-\tilde{g}$, $h_{2}=\tilde{g}-g_{0}$, we have

(58)
$$\;|\frac{1}{n}\sum_{i=1}^{n}\left\{e^{-\tilde{g}(X_{i})}-e^{-g_{0}(X_{i})}\right\}(\hat{g}-\tilde{g})(X_{i})+V^{*}(\hat{g}-\tilde{g},\tilde{g}-g_{0})|\;\leq |V^{*}(\hat{g}-\tilde{g},\tilde{g}-g_{0})-\frac{1}{n}\sum_{i=1}^{n}e^{-g_{0}(X_{i})}(\hat{g}-\tilde{g})(X_{i})(\tilde{g}-g_{0})(X_{i})|\;\;+|\frac{1}{n}\sum_{i=1}^{n}e^{-g_{0}(X_{i})}(\hat{g}-\tilde{g})(X_{i})(\tilde{g}-g_{0})(X_{i})-\frac{1}{n}\sum_{i=1}^{n}c_{0}^{\left(i\right)}e^{-g_{0}(X_{i})}(\hat{g}-\tilde{g})(X_{i})(\tilde{g}-g_{0})(X_{i})|\;\leq (o_{p}(1)+2c_{0}){\left\{(V^{*}+\lambda_{1}J^{*})(\hat{g}-\tilde{g})(V^{*}+\lambda_{1}J^{*})(\tilde{g}-g_{0})\right\}}^{1∕2},$$

where the first inequality follows (54) and the second inequality follows Lemma 2 and 3. Combining (52), (57), and (58), we obtain

(59)
$$(\frac{c_{2}}{2}V^{*}+\lambda_{1}J^{*})(\hat{g}-\tilde{g})\leq (o_{p}(1)+2c_{0}){\left\{(V^{*}+\lambda_{1}J^{*})(\hat{g}-\tilde{g})(V^{*}+\lambda_{1}J^{*})(\tilde{g}-g_{0})\right\}}^{1∕2}.$$

Combining (59) with Lemma 1, as $\lambda_{1}\to 0$ and $n\lambda_{1}^{2∕r}\to \infty$, we have $\left(V^{*}+\lambda_{1}J^{*})(\hat{g}-\tilde{g})=O_{p}(n^{-1}\lambda_{1}^{-1∕r}+\lambda_{1}^{l}\right)$ and Theorem 1 holds. □

**Proof of** Theorem 2: We know

(60)
$$\sum_{k\neq \alpha }{\left\|\eta_{\alpha k}(x_{\alpha },x_{k})\right\|}^{2}\leq {\left\{\sum_{k\neq \alpha }\|\eta_{\alpha k}(x_{j},x_{k})\|\right\}}^{2}\leq (p-1)\sum_{k\neq \alpha }{\left\|\eta_{\alpha k}(x_{\alpha },x_{k})\right\|}^{2}.$$

Therefore, there exists some constant $C\in [1,\sqrt{p-1}]$ such that $C{\left\{\sum_{k\neq \alpha }{\left\|\eta_{\alpha k}(x_{\alpha },x_{k})\right\|}^{2}\right\}}^{1∕2}=\sum_{k\neq \alpha }\|\eta_{\alpha k}(x_{\alpha },x_{k})\|$. Since $\sum_{k\neq \alpha }\theta_{\alpha k}$ is bounded by M, we can scale $\lambda_{1}$ and $\lambda_{2}$ such that $\theta_{\alpha k}\leq 1$. Since $J_{2}^{*}(g)=\sum_{k\neq \alpha }\theta_{\alpha k}^{-1}{\left\|\eta_{\alpha k}(x_{\alpha },x_{k})\right\|}^{2}=c^{T}(\sum_{k\neq \alpha }\theta_{\alpha k}Q_{\alpha k})c,\sum_{k\neq \alpha }{\left\|\eta_{\alpha k}(x_{\alpha },x_{k})\right\|}^{2}=c^{T}(\sum_{k\neq \alpha }\theta_{\alpha k}^{2}Q_{\alpha k})c$, we have $J_{2}^{2}(g)=C^{2}\sum_{k\neq \alpha }{\left\|\eta_{\alpha k}(x_{\alpha },x_{k})\right\|}^{2}\leq C^{2}J_{2}^{*}(g)$ and consequently $J_{2}\leq C{\left(J^{*}\right)}^{1∕2}$. Furthermore, since $V_{2}^{2}(g^{\left(2\right)})=\int_{{𝒳}_{\setminus \{\alpha \}}}f_{\setminus \{\alpha \}}(x_{\setminus \{\alpha \}})\int_{{𝒳}_{\alpha }}{\left\{g^{\left(2\right)}(x)\right\}}^{2}\rho (x)dx_{\alpha }dx_{\setminus \{\alpha \}}=V^{*}(g^{\left(2\right)})$, we have $V_{2}(g^{\left(2\right)})={\left[V^{*}(g^{\left(2\right)})\right]}^{1∕2}$. Therefore, $\left(V_{2}+\lambda_{1}J_{2})(g^{\left(2\right)})=({\left(V^{*}\right)}^{1∕2}+C\sqrt{\lambda_{1}}{\left(\lambda_{1}J^{*}\right)}^{1∕2})(g^{\left(2\right)})\leq {\left(1+C^{2}\lambda_{1}\right)}^{1∕2}{\left(V^{*}+\lambda_{1}J^{*}\right)}^{1∕2}(g^{\left(2\right)}\right)$ by the Cauchy-Schwarz inequality. Finally,

(61)
$$(V+\lambda_{1}J)(\hat{g}-g^{0})=(V_{1}+\lambda_{1}J_{1})({\hat{g}}^{\left(1\right)}-g_{0}^{\left(1\right)})+(V_{2}+\lambda_{1}J_{2})({\hat{g}}^{\left(2\right)}-g_{0}^{\left(2\right)})\;\;\leq (V^{*}+\lambda_{1}J^{*})({\hat{g}}^{\left(1\right)}-g_{0}^{\left(1\right)})+{\left(1+C^{2}\lambda_{1}\right)}^{1∕2}{\left(V^{*}+\lambda_{1}J^{*}\right)}^{1∕2}({\hat{g}}^{\left(2\right)}-g_{0}^{\left(2\right)})\;\;=O_{p}(n^{-1}\lambda_{1}^{-1∕r}+\lambda_{1}^{l})+O(n^{-1∕2}\lambda_{1}^{-1∕2r}+\lambda_{1}^{l∕2})\;\;=O_{p}(n^{-1∕2}\lambda_{1}^{-1∕2r}+\lambda_{1}^{l∕2}).$$

□

**Proof of** Corollary 1: By the definition of V(⋅), $V(\hat{g}-g^{0})=V_{1}({\hat{g}}^{\left(1\right)}-g_{0}^{\left(1\right)})+V_{2}({\hat{g}}^{\left(2\right)}-g_{0}^{\left(2\right)})=V^{*}({\hat{g}}^{\left(1\right)}-g_{0}^{\left(1\right)})+{\left[V^{*}({\hat{g}}^{\left(2\right)}-g_{0}^{\left(2\right)})\right]}^{1∕2}$. Following (61),

$${\left[V^{*}({\hat{g}}^{\left(2\right)}-g_{0}^{\left(2\right)})\right]}^{1∕2}=O_{p}(n^{-1∕2}\lambda_{1}^{-1∕2r}+\lambda_{1}^{l∕2}).$$

Following Lin et al. (2000), under the condition $0<c_{5}<\rho (x)<c_{6}$ and $0<c_{7}<f_{\setminus \{\alpha \}}(x_{\setminus \{\alpha \}})<c_{8}$ for some positive constants $c_{5}$, $c_{6}$, $c_{7}$, and $c_{8}$, ${\left[V^{*}(g)\right]}^{1∕2}$ is equivalent to the $L_{2}$ norm. Specifically, $V^{*}(g)∼{\left\|g\right\|}_{2}^{2}=\sum_{j=1}^{p}{\left\|\eta_{j}\right\|}_{2}^{2}+\sum_{k\neq \alpha }{\left\|\eta_{\alpha k}(x_{\alpha },x_{k})\right\|}_{2}^{2}$, $V^{*}(g^{\left(1\right)})∼\sum_{j=1}^{p}{\left\|\eta_{j}\right\|}_{2}^{2}$, and $V^{*}(g^{\left(2\right)})∼\sum_{k\neq \alpha }{\left\|\eta_{\alpha k}(x_{\alpha },x_{k})\right\|}_{2}^{2}$, where ∼ means equivalence. By definition, $V(g^{\left(2\right)})={\left[V^{*}(g^{\left(2\right)})\right]}^{1∕2}∼{\left(\sum_{k\neq \alpha }{\left\|\eta_{\alpha k}(x_{\alpha },x_{k})\right\|}_{2}^{2}\right)}^{1∕2}$. Consequently, two-way interactions under $L_{2}$ norm have the same convergence rate as ${\left[V^{*}(g^{\left(2\right)})\right]}^{1∕2}$,

$${\left\|{\hat{\eta }}_{\alpha k}-\eta_{0\alpha k}\right\|}_{2}=O_{p}(n^{-1∕2}\lambda_{1}^{-1∕2r}+\lambda_{1}^{l∕2}),\;\;k\neq \alpha ,\;k=1,\cdots ,p.$$

□
